# A global database for metacommunity ecology, integrating species, traits, environment and space

**DOI:** 10.1038/s41597-019-0344-7

**Published:** 2020-01-08

**Authors:** Alienor Jeliazkov, Darko Mijatovic, Stéphane Chantepie, Nigel Andrew, Raphaël Arlettaz, Luc Barbaro, Nadia Barsoum, Alena Bartonova, Elena Belskaya, Núria Bonada, Anik Brind’Amour, Rodrigo Carvalho, Helena Castro, Damian Chmura, Philippe Choler, Karen Chong-Seng, Daniel Cleary, Anouk Cormont, William Cornwell, Ramiro de Campos, Nicole de Voogd, Sylvain Doledec, Joshua Drew, Frank Dziock, Anthony Eallonardo, Melanie J. Edgar, Fábio Farneda, Domingo Flores Hernandez, Cédric Frenette-Dussault, Guillaume Fried, Belinda Gallardo, Heloise Gibb, Thiago Gonçalves-Souza, Janet Higuti, Jean-Yves Humbert, Boris R. Krasnov, Eric Le Saux, Zoe Lindo, Adria Lopez-Baucells, Elizabeth Lowe, Bryndis Marteinsdottir, Koen Martens, Peter Meffert, Andres Mellado-Díaz, Myles H. M. Menz, Christoph F. J. Meyer, Julia Ramos Miranda, David Mouillot, Alessandro Ossola, Robin Pakeman, Sandrine Pavoine, Burak Pekin, Joan Pino, Arnaud Pocheville, Francesco Pomati, Peter Poschlod, Honor C. Prentice, Oliver Purschke, Valerie Raevel, Triin Reitalu, Willem Renema, Ignacio Ribera, Natalie Robinson, Bjorn Robroek, Ricardo Rocha, Sen-Her Shieh, Rebecca Spake, Monika Staniaszek-Kik, Michal Stanko, Francisco Leonardo Tejerina-Garro, Cajo ter Braak, Mark C. Urban, Roel van Klink, Sébastien Villéger, Ruut Wegman, Martin J. Westgate, Jonas Wolff, Jan Żarnowiec, Maxim Zolotarev, Jonathan M. Chase

**Affiliations:** 1 German Centre for Integrative Biodiversity Research (iDiv), Deutscher Platz 5E, 04103 Leipzig, Germany; 2 Department of Computer Science, Martin Luther University Halle-Wittenberg, 06099 Halle, Salle Germany; 3 Department of Evolutionary Biology and Environmental Studies, University of Zürich, Winterthurerstrasse 190, 8057 Zurich, Switzerland; 4 Zoology, University of New England, Armidale, NSW 2351 Australia; 5 Division of Conservation Biology, Institute of Ecology and Evolution, University of Bern, 3012 Bern, Switzerland; 6 Dynafor, INRA-INPT, Univ. Toulouse, Auzeville, France; 7 Centre d’Ecologie et des Sciences de la Conservation (CESCO), Muséum National d’Histoire Naturelle, CNRS, Sorbonne Université, Paris, France; 8 Centre for Ecosystems, Society and Biosecurity, Forest Research, Alice Holt Lodge, Farnham, Surrey GU10 4LH UK; 9 Biology Centre CAS, Czech Academy of Sciences, Institute of Entomology, Branisovska 31, 370 05 Ceske Budejovice, Czech Republic; 10 University of South Bohemia in Ceske Budejovice, Faculty of Science, Branisovska 1760, 370 05 Ceske Budejovice, Czech Republic; 11 Institute of Plant and Animal Ecology, Ural Branch, Russian Academy of Sciences, Eighth March Street 202, Yekaterinburg, 620144 Russia; 12 Grup de Recerca “Freshwater Ecology, Hydrology and Management” (FEHM), Departament de Biologia Evolutiva, Ecologia i Ciències Ambientals, Facultat de Biologia, Institut de Recerca de la Biodiversitat (IRBio), Universitat de Barcelona (UB), Diagonal 643, 08028 Barcelona, Catalonia Spain; 13 Unité Écologie et Modèles pour l’Halieutique, IFREMER, Rue de l’île d’Yeu, B.P. 21105, 44311 Nantes, Cedex 03 France; 14 Departamento de Biologia, Universidade Estadual de Goiás, Campus Palmeiras de Goiás, Palmeiras de Goiás, Goiás, Brazil; 15 Universidade Estadual de Goiás (UEG), Programa de Pós-Graduação em Recursos Naturais do Cerrado (RENAC), Campus de Ciências Exatas e Tecnológicas - Henrique Santillo, BR 153, No. 3105 Fazenda Barreiro do Meio, 75132400 Anápolis, GO Brazil; 16 CFE- Centre for Functional Ecology - Science for People & the Planet, Department of Life Sciences, University of Coimbra, 3000-456 Coimbra, Portugal; 17 Institute of Environmental Protection and Engineering, University of Bielsko-Biala, Willowa 2, 43-309 Bielsko-Biała, Poland; 18 Univ. Grenoble Alpes, Univ. Savoie Mont Blanc, CNRS, LECA, F-38000 Grenoble, France; 19 ARC Centre of Excellence for Coral Reef Studies, James Cook University, Townsville, QLD 4811 Australia; 20 Department of Biology & CESAM, University of Aveiro, Campus de Santiago, 3810-193 Aveiro, Portugal; 21 Tropical Island Sustainable Development Research Center, National Penghu University of Science and Technology, 300 Liu-Ho Rd., Magong City, Penghu 880 Taiwan; 22 Wageningen Environmental Research, Wageningen University & Research, Droevendaalsesteeg 3-3 A, 6708 PB Wageningen, The Netherlands; 23 Ecology and Evolution Research Centre, School of Biological, Earth and Environmental Sciences, UNSW Sydney, Sydney, New South Wales 2052 Australia; 24 Programa de Pós-Graduação em Ecologia de Ambientes Aquáticos Continentais, Núcleo de Pesquisas em Limnologia, Ictiologia, Universidade Estadual de Maringá. Av. Colombo, 5790, CEP, 87020-900 Maringá, PR Brazil; 25 Naturalis Biodiversity Center, Marine Biodiversity, Vondellaan 55, 2332 AA Leiden, The Netherlands; 26 Institute of Environmental Sciences, Environmental Biology Department, Leiden University, Einsteinweg 2, 2333 CC Leiden, The Netherlands; 27 UMR 5023 LEHNA, Université Lyon 1, Université Lyon, Villeurbanne, France; 28 Department of Environmental and Forest Biology. State University of New York, College of Environmental Science and Forestry. 1 Forestry Dr. Syracuse, New York, NY 13210 USA; 29 University of Applied Sciences HTW Dresden, Pillnitzer Platz 2, D-01326 Dresden, Germany; 30 OBG, Part of Ramboll, 400 Andrews St., Suite 710, Rochester, NY 14604 USA; 31 Department of Life Sciences, Natural History Museum, Cromwell Road, London, SW7 5BD UK; 32 Department of Ecology, Federal University of Rio de Janeiro, 21941-902 Rio de Janeiro, Brazil; 33 Biological Dynamics of Forest Fragments Project, National Institute for Amazonian Research and Smithsonian Tropical Research Institute, 69011-970 Manaus, Brazil; 34 Centre for Ecology, Evolution and Environmental Changes, University of Lisbon, 1749-016 Lisbon, Portugal; 35 Instituto EPOMEX, Universidad Autónoma de Campeche, Av. Héroe de Nacozari No. 480, Campus VI de Investigación-UAC, San Francisco de Campeche, 24020 Campeche, México; 36 Institut de recherche en biologie végétale, Montréal, Québec, Canada; 37 Anses, Laboratoire de la Santé des Végétaux, Unité Entomologie et Plantes Invasives, Montferrier-sur-Lez, France; 38 Pyrenean Institute of Ecology (IPE-CSIC). Avda. Montanana, 1005 zaragoza, Spain; 39 Department of Ecology, Environment and Evolution and Centre for Future Landscapes, La Trobe University, Melbourne, Victoria 3086 Australia; 40 Department of Biology, Ecological Synthesis and Biodiversity Conservation Lab, Federal Rural University of Pernambuco, Rio de Janeiro, Brazil; 41 Mitrani Department of Desert Ecology, Swiss Institute for Dryland Environmental and Energy Research, Jacob Blaustein Institutes for Desert Research, Ben-Gurion University of the Negev, Sede-Boqer Campus, Midreshet Ben-Gurion, 8499000 Israel; 42 Department of Biology, The University of Western Ontario, London, Ontario Canada; 43 Granollers Museum of Natural Sciences, 08402 Granollers, Catalonia Spain; 44 Department of Biological Sciences, Macquarie University, Sydney, NSW 2109 Australia; 45 The soil conservation service of Iceland, Gunnarsholt, 851 Hella, Iceland; 46 Royal Belgian Institute of Natural Sciences, Vautierstraat 29, 1000 Brussels, Belgium; 47 University of Ghent, Department of Biology, K.L. Ledeganckstraat 35, 9000 Ghent, Belgium; 48 corvus, Lüchow 2, D-17179 Altkalen, Germany; 49 Departamento de Ecología e Hidrología, Facultad de Biología, Universidad de Murcia, 30100 Murcia, Spain; 50 Present Address: Gerencia de Planificación y Gestión Hídrica. TRAGSATEC. C/Valentín Beato, 6, 28037 Madrid, Spain; 51 Department of Migration and Immuno-ecology, Max Planck Institute for Ornithology, 78315 Radolfzell, Germany; 52 School of Environment and Life Sciences, University of Salford, M5 4WT Salford, UK; 53 MARBEC, Univ Montpellier, CNRS, Ifremer, IRD, Montpellier, France; 54 The James Hutton Institute, Craigiebuckler, Aberdeen, AB15 8QH UK; 55 Istanbul Technical University, Eurasia Institute of Earth Sciences, Istanbul, 34469 Turkey; 56 CREAF, Cerdanyola del Vallés 08193, Spain/UAB, Cerdanyola del Vallés, 08193 Spain; 57 CNRS & Université Paul Sabatier, Laboratoire Évolution et Diversité Biologique, UMR 5174, Bât. 4R1, 118 Route de Narbonne, F-31062 Toulouse, cedex 9 France; 58 Eawag, Swiss Federal Institute of Water Science and Technology, Uberlandstrasse 133, 8600 Dubendorf, Switzerland; 59 Ecology and Conservation Biology, Institute for Plant Sciences, University of Regensburg, D-93040 Regensburg, Germany; 60 Department of Biology, Lund University, Sölvegatan 37, SE 223 62 Lund, Sweden; 61 CEFE UMR 5175, CNRS - Université de Montpellier - Université Paul-Valéry Montpellier - EPHE, 1919 route de Mende, F-34293 Montpellier, Cedex 5 France; 62 Institute of Geology, Tallinn University of Technology, Ehitajate tee 5, 19086 Tallinn, Estonia; 63 Institute of Evolutionary Biology (CSIC-Universitat Pompeu Fabra), Passeig Maritim Barceloneta 37, 08003 Barcelona, Spain; 64 National Ecological Observatory Network, 1685 38th Street Suite 100, Boulder, CO 80301 USA; 65 University of Colorado Department of Ecology and Evolutionary Biology, UCB 334, University of Colorado, Boulder, CO 80309 USA; 66 School of Biological Sciences, University of Southampton, Highfield Campus, Southampton, SO17 1BJ UK; 67 Department of Ecological Humanities, Providence University, 200, Sec. 7, Taiwan Boulevard, Shalu Dist., Taichung 43301 Taiwan; 68 School of Geography and Environmental Science, University of Southampton, Highfield Campus, Southampton, SO17 1BJ UK; 69 Department of Geobotany and Plant Ecology, University of Lodz, Banacha 12/16, 90-237 Łódź, Poland; 70 Institute of Parasitology and Institute of Zoology, Slovak Acad. Sci., Loffl erova 10, SK-04001 Kosice, Slovakia; 71 Centro de Biologia Aquática, Escola de Ciências Agrárias e Biológicas, Pontifícia Universidade Católica de Goiás, Campus II. Av. Engler s/n, Jd. Mariliza, Goiânia, Goiás CEP 74885460 Brazil; 72 Laboratório de Biodiversidade, Programa de Pós-Graduação em Sociedade Tecnologia e Meio Ambiente, UniEVANGÉLICA. Avenida Universitária km 3,5, Cidade Universitária, Anápolis, Goiás CEP 75083-515 Brazil; 73 Biometris, Wageningen University & Research, Droevendaalsesteeg 1, 6708 PB Wageningen, The Netherlands; 74 University of Connecticut, 75 N. Eagleville Road, Unit 3043, Storrs, CT 06269 USA; 75 Fenner School of Environment & Society, The Australian National University, Acton, ACT 2601 Australia

**Keywords:** Community ecology, Macroecology, Biodiversity

## Abstract

The use of functional information in the form of species traits plays an important role in explaining biodiversity patterns and responses to environmental changes. Although relationships between species composition, their traits, and the environment have been extensively studied on a case-by-case basis, results are variable, and it remains unclear how generalizable these relationships are across ecosystems, taxa and spatial scales. To address this gap, we collated 80 datasets from trait-based studies into a global database for *metaCommunity Ecology: Species, Traits, Environment and Space*; “CESTES”. Each dataset includes four matrices: species community abundances or presences/absences across multiple sites, species trait information, environmental variables and spatial coordinates of the sampling sites. The CESTES database is a live database: it will be maintained and expanded in the future as new datasets become available. By its harmonized structure, and the diversity of ecosystem types, taxonomic groups, and spatial scales it covers, the CESTES database provides an important opportunity for synthetic trait-based research in community ecology.

## Abstract (summary)

**Table:** 

Measurement(s)	species abundance • species trait • environmental feature • latitude • longitude
Technology Type(s)	digital curation
Factor Type(s)	year of data collection • type of ecosystem • level of human disturbance
Sample Characteristic - Environment	terrestrial natural environment • fresh water • marine biome • anthropogenic terrestrial biome • natural environment • area of mixed forest • cultivated environment
Sample Characteristic - Location	Earth (planet)

Machine-accessible metadata file describing the reported data: 10.6084/m9.figshare.11317790

## Background & Summary

A major challenge in ecology is to understand the processes underlying community assembly and biodiversity patterns across space^1,2^. Over the three last decades, trait-based research, by taking up this challenge, has drawn increasing interest^3^, in particular with the aim of predicting biodiversity response to environment. In community ecology, it has been equated to the ‘*Holy Grail’* that would allow ecologists to approach the potential processes underlying metacommunity patterns^4–7^. In macroecology, it is common to study biodiversity variation through its taxonomic and functional facets along gradients of environmental drivers^8–10^. In biodiversity-ecosystem functioning research, trait-based diversity measures complement taxonomic ones to predict ecosystem functions^11^ offering early-warning signs of ecosystem perturbation^12^.

The topic of Trait-Environment Relationships (TER) has been extensively studied across the globe and across the tree of life. However, each study deals with a specific system, taxonomic group, and geographic region and uses different methods to assess the relationship between species traits and the environment. As a consequence, we do not know how generalizable apparent relationships are, nor how they vary across ecosystems, realms, and taxonomic groups. In addition, while there is an emerging synthesis about the role of traits for terrestrial plant communities^13,14^, we know much less about other groups and ecosystem types.

**Fig. 1:**
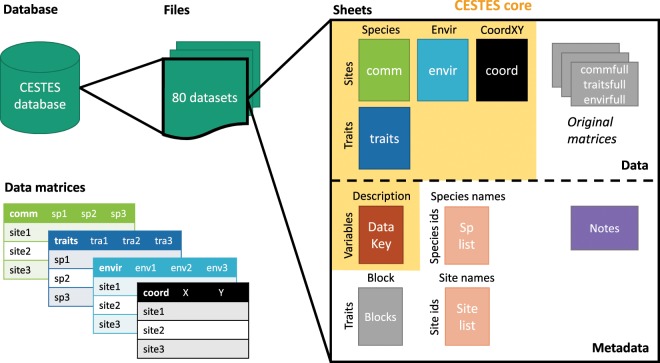
Structure of the CESTES database. The database includes 80 Excel files for 80 datasets. Each dataset is composed of four matrices of data stored in spreadsheets: comm (species abundances [n = 68] or presences/absences [n = 12]), traits (species traits), envir (environmental variables), and coord (spatial coordinates). Each dataset also includes a DataKey (description of the entries of the Data tables), a Notes sheet (contact information for the dataset, and, when relevant, processing information), a Species list, and a Site list. The grey components can be the original data matrices, and additional information and do not appear in all the datasets, depending on specific needs (see Methods - Data processing section).

**Fig. 2:**
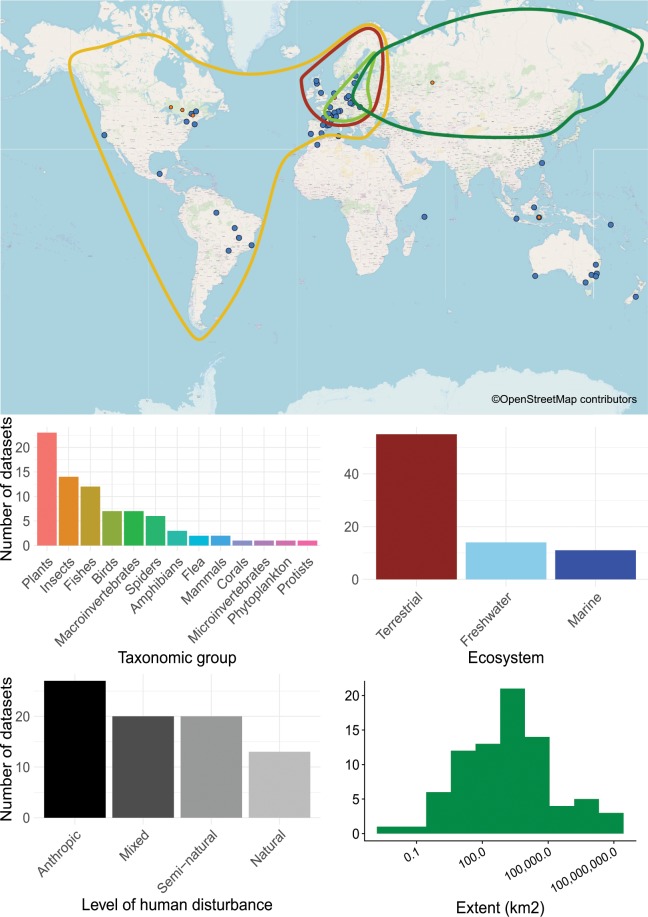
Overview of the CESTES database. Upper panel: Map of the 80 dataset locations over the globe (blue spots) (the orange smaller spots represent the 10 ancillary datasets from *ceste*, the non-spatial supplement of CESTES - see the Methods section); the four coloured polygons represent four datasets that are covering continental extents. The background world map is from OpenStreetMap contributors. Bottom panel: Bar plots and histogram describing the content of the database in terms of: study group, ecological realm, level of human disturbance, and spatial extent of the study.

To address these gaps, we introduce the CESTES database - a global database for *metaCommunity Ecology: Species, Traits, Environment and Space*. This database assembles 80 datasets from studies that analysed empirical multivariate trait-environment relationships between 1996 (the first multivariate study of TER^15^) and 2018. All considered datasets include four data matrices (Fig. 1): (i) community data (species abundances or presences/absences across multiple sites), (ii) species traits (*sensu lato*), (iii) environmental variables across sites, and (iv) spatial coordinates. The database is global in extent and covers different taxonomic groups, ecosystem types, levels of human disturbance, and spatial scales (Fig. 2).

Several global trait databases already exist or are emerging, such as the Open Traits working group^16^, the Freshwater Information Platform and its Taxa and Autecology Database for Freshwater Organisms^17^, the PREDICTS database for Projecting Responses of Ecological Diversity In Changing Terrestrial Systems^18,19^, and the TRY^20^ plant trait database for Quantifying and scaling global plant trait diversity. In comparison to these initiatives, the CESTES database has several unique features. Specifically, it maintains the original matching between the community, environmental, and spatial data that go along with the trait information. Keeping this original matching of the data ensures homogeneity in the data structure and allows for targeted analyses of TER. We include all taxonomic groups for which the appropriate matrices are available including groups poorly represented in most trait compilations (e.g., invertebrates and bats). The trait information is particularly diverse, ranging from life-history and morphological to trophic traits, dispersal abilities and tolerances, and covering various ecological mechanisms. CESTES only includes data where georeferenced coordinates, or relative coordinates of the sampling sites (hereafter: spatial coordinates) and environmental variables are available to enable spatial and scaling community analyses. We prioritized studies with abundance or biomass data (as opposed to presence/absence) to facilitate the calculation of a broad range of biodiversity metrics and the study of different facets of biodiversity. The data available in CESTES are open access without restriction, except via citation of this paper (and any original paper that plays a particularly important role in the analyses). Importantly, the CESTES database is meant to be a live database^21^: it will be maintained in the future and new datasets will be added as they become available.

The CESTES database aims to significantly contribute to research in biogeography, macroecology (including in complement with phylogenies), community and metacommunity ecology, and biodiversity-ecosystem functioning. On the one hand, the quality of its content and structure will allow meta-analyses and syntheses (e.g., the role of taxonomic and functional diversity in spatial patterns of communities). On the other hand, specific datasets will enable the exploration of new questions on a given group, realm, or type of ecosystem.

## Methods

### Data compilation

#### Database scoping

The rationale for developing the CESTES database is generally for the study of TER in relation to metacommunity ecology and/or macroecological questions. As such, we focussed on datasets that were appropriate within the metacommunity or macroecology context (i.e. species assemblages distributed across space) and that focussed on traits to understand biodiversity patterns and responses. This prerequisite led us to identify multivariate trait-based studies as the most relevant and rich source of datasets that could fulfil these two requirements.

Given the complexity that still pertains to trait typology^13^, we did not restrict ourselves to any specific definition of traits and integrated all possible species characteristics if they were used as “traits” in the original study. We thus included ecophysiological, functional, life-history and biological traits, as well as response and effects traits. CESTES users can select traits according to their study needs.

We identified eligible datasets based on two strategies: 1. Literature search, aiming to initiate the database construction along a structured workflow, 2. Networking, aiming to extend the database and open the sharing possibilities, if the datasets fulfilled the CESTES requirements.

The main condition for dataset eligibility was that the TER was the focus of the study and data use. This ensured that: 1. the trait and the taxonomic information were collected from similar biogeographic areas (minimizing mismatches between the geographic origins of trait and taxonomic data), 2. the sampled sites were associated with contextual environmental information that was relevant to the community and traits under study.

#### Literature search

We searched for multivariate trait-based studies published between 1996 and 2018 via a systematic literature search on the Clarivate Analytics Web of Science Core database. Following Leibold & Chase^2^, we focussed on studies that included (in any of their contents) the following terms (including spelling variations): “RLQ”^15^ and “fourth-corner”^22,23^ because both of them are the predominant methods of multivariate trait-based analyses in ecology^24^. The “RLQ” refers to a co-inertia analysis that summarizes the overall link between the three matrices of species abundances/presences-absences (L), species traits (Q) and environment (R). The “fourth-corner” refers to a permutation analysis of these three matrices that tests individual trait-environment relationships. The use of RLQ and fourth-corner analyses on the datasets ensures that all of them: 1. are multivariate and include both several species, several traits, and several sites (potentially including spatial information) to align with a metacommunity-like structure, 2. have a comparable structure and can be used in comparative analyses and syntheses.

The search query was:

*ALL* = (“*fourth-corner*” *OR* “*fourth corner*” *OR* “*fourthcorner*” *OR* “*RLQ*”)

This search resulted in 368 papers.

Note that the “fourth corner” term more generally and commonly refers to the widely studied question of the links between trait and environment variations^22^. Most studies that look at TER, regardless of the method of analysis they use, would often acknowledge the historical background of their question by referring in their paper to the “fourth corner problem”. Consequently, by including the “fourth corner” search term, we identified eligible multivariate datasets that were not necessarily analysed by fourth corner analysis/RLQ, but also by e.g. trait-based generalized linear/additive models^25,26^. However, although this literature search strategy was well suited for identifying sources of multivariate datasets, it could appear as too specific. In order to relax the constraints due to this specificity, we complemented the data search by a networking strategy (see *Networking* section).

#### Scanning strategy

Among the 368 studies resulting from the literature search, we scanned through the Introduction and Methods sections. We selected the studies that used at least the three matrices of species abundances, or presences/absences across multiples sites (“comm”), corresponding environment information across sites (“envir”), and species trait information (“traits”). At first, we prioritized datasets that had spatial coordinates of the sampling sites (“coord”) because the spatial aspect is crucial for metacommunity research^2^. Spatial coordinates, or the relative locations, could sometimes be reconstructed from the maps presented in the publications. Review and opinion papers, medical and simulation studies were not considered. Following this filter, we identified a subset of 105 eligible datasets.

#### Networking

The network strategy took place in parallel to the data search and relied on both formal and informal communications and exchanges with colleagues through conferences, workshops, group meetings, emails, etc. This allowed us to identify new data providers, or new datasets that we had not found via the earlier literature search. From this networking, we identified an additional set of 34 potentially eligible datasets.

#### Dataset collection and request

From the total of 139 eligible datasets, 7.2% of the datasets were available on the online supplementary materials of the publication. These were downloaded and formatted for CESTES’ purposes.

**Fig. 3:**
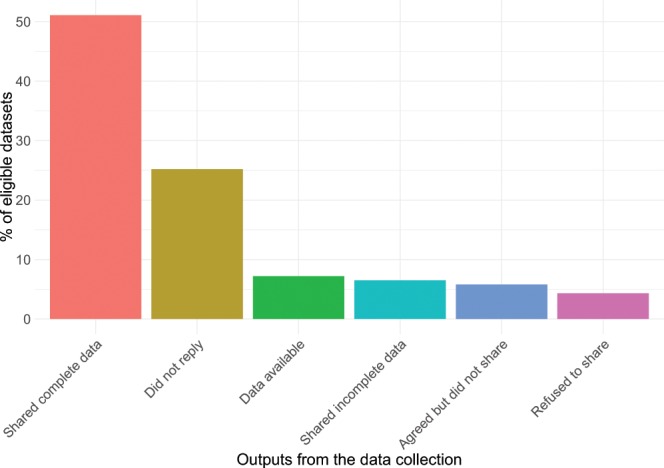
Success rates of the data search and request. Barplot showing the percentage of the different outputs from the data collection process. Percentages are calculated from a total of 139 datasets identified as eligible for the CESTES database (based on literature search and networking). Incomplete data mainly refer to the datasets that had no spatial coordinates (*ceste*), included unsolved issues, or provided insufficient metadata information. (“Agreed but did not share” refers to authors who replied positively to the first request but then never sent their data despite reminders because e.g., they did not find time to prepare the data).

When the datasets were not directly available, we sent a data request via email. In order to launch the CESTES database in a reasonable amount of time, we had to set time limits for the request phase, namely between January and August 2018. As a result, in total 96 authors were contacted, of whom 58% shared their data. In terms of datasets, more than 50% of the eligible datasets were shared and complete (Fig. 3). We also received ‘spontaneous’ datasets that were not part of our initial request, but fulfilled CESTES’ requirements and were thus included in the database. Out of the final complete 80 datasets, 55 were obtained via the literature search, and 25 were obtained from the networking strategy.

Because we received 10 valuable datasets that had no spatial coordinates, we decided to open the *ceste* subsection of the CESTES database and populate it with these specific datasets. Some of them could be upgraded to CESTES database when the authors are able to provide the coordinates.

### Data processing

#### Dataset checking, cleaning and formatting

We downloaded and received datasets in various formats (.doc, .pdf, .csv, .RData, .txt, .shp, etc.). Following Broman & Woo^27^, we harmonized and gathered them in Excel files, one file per dataset. This was the most convenient storage format for creating multiple sheets (community, traits, environment, coordinates), handling heterogeneous types of information, and building metadata specific to each dataset. This storage solution also facilitated visual checking and cleaning of the data records.

CESTES provides both the processed and the unprocessed (i.e. original) datasets. The *processed* datasets include “comm”, “traits” and “envir”, i.e. with no empty sites, no “ghost” species (i.e. species that are recorded in none of the sites of the study area), and no NAs (Not Available information) in the matrices. NA removal was based on a compromise in the relative frequency of NAs in the rows and columns of each table; when too many sites compared to the sample size (e.g. >50% of the sites) had NAs for one single variable, this variable was removed, whereas when there were some sites (e.g. <30% of the sites) showing NAs for more than one variable, we removed those sites instead of removing the variables. Since CESTES is primarily designed for trait-based analyses, we removed a trait when it included too many NAs across species (i.e. when the trait value was NA for more than 50% of the species in the community). Similarly, we removed species for which no, or too incomplete trait information was available (i.e. when keeping the species would have implied to lose several traits). This was the case for 29 datasets out of the 80. The number of species removed varied from 1 to 209 species (mean = 27, median = 10, sd = 45) that represented from 1 to 72% of the initial species pool (mean = sd = 17%). (Note that this high maximum value is due to only one single dataset where trait data were exceptionally limiting and implied to remove an important number of species without trait information).

When this overall cleaning procedure implied removing any of the species, traits, or environmental variables, we kept the information of the original *unprocessed* tables within the Excel file in separate sheets. We named these sheets “commfull”, “traitsfull” and “envirfull”, respectively. Thus, the user can either directly use the processed sheets (“comm”, “traits” and “envir”), or the original ones and apply any other filtering strategies. In doing so, we make sure that CESTES is flexible depending on the users’ goals and needs.

Cleaning steps that altered the original dataset (other than formatting) are reported in the “Notes” sheet so that the user can trace back what has been done over the data processing.

When the data included several temporal horizons (sampling years, or seasons treated as different replicates in the original publication), we split them into different datasets for each time horizon to facilitate further analyses. This explains why several datasets can correspond to one single study area (see Online-only Table 1 attached to this manuscript, and the Data Records section).

#### Metadata preparation

**Fig. 4:**
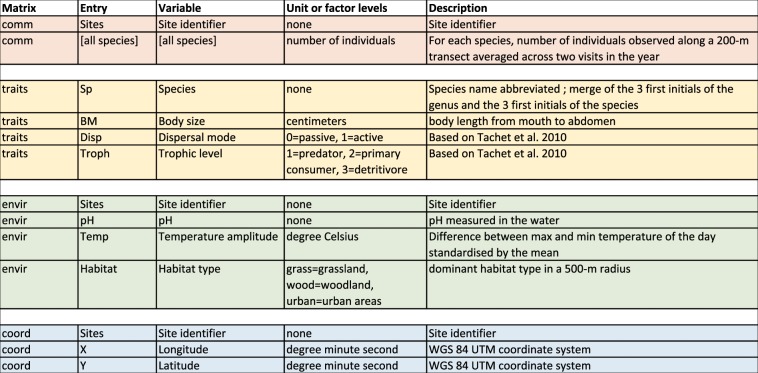
“DataKey” structure and example of metadata information in CESTES datasets. A description is given when the variable full name is not self-explanatory or when potentially relevant information was available. Possible empty cells are due to lack of information that could not be recovered from the original publication nor from the data owners.

All the entries from the four data sheets - “comm”, “trait”, “envir” and “coord” - were listed and described in a “DataKey” sheet to describe the tables’ content (Fig. 4). This required a thorough examination of the original papers to extract the relevant information for every dataset. In several cases, we required additional exchanges with the data owners for clarifications. Any empty cell in the “DataKey” sheet reflects a lack of information. Importantly, this sheet should not substitute for reading of the original paper and we strongly recommend the users to thoroughly examine each paper before using the data (see Online-only Table 2).

## Data Records

### Storage and formats used

We stored the CESTES database via three different storage systems and two types of formats to provide the users with several alternatives in accessing and using the data.

Storage

- Figshare repository: 10.6084/m9.figshare.c.4459637 ^28^ -> the **fixed version** of the database, and R scripts (original version 1.0).
- iDiv Biodiversity Portal: 10.25829/idiv.286-21-2695 ^29^ under the *Primary Data* tab -> the **upgraded versions** of the database, and R scripts following the updates when new datasets are integrated.

Formats

A zipped folder called “CESTES.zip” includes two alternative formats for the CESTES database:

- a “xCESTES” folder that includes 80 Excel files (one file per dataset), each named according to the following structure: “AuthorPublicationYear.xlsx”.
- a “rCESTES” folder that includes the CESTES core processed database (comm, traits, envir, coord matrices) as an R list object “CESTES.RData” plus two R scripts, and two metadata tables for data processing and exploration (see Usage Notes section).
  The “CESTES.zip” folder also includes:
- an extended metadata table, “CESTES_metadata.xlsx”, that provides the general metadata information of all the datasets (i.e., combining the information from the Online-only Tables 1–2 of this Data Descriptor)
- a tutorial document, “HOW_TO_SHARE_MY_DATA_FOR_CESTES.pdf”, that explains how to share data for integrating future datasets in the database (see Supplementary File 1).

The original, unprocessed files as they were provided by the data owners (thus possibly in different formats, various structures, with possible mistakes, without metadata, etc.), are available by request to the corresponding author, AJ.

We would also encourage any potential data contributors to contact AJ with possible data (cestes@idiv.de). The specific guidelines on which types of data are eligible to integration in CESTES, and on how to structure the data, and metadata are provided in the Supplementary File 1, as well as at: https://icestes.github.io/sharedata. If the dataset fulfils CESTES’ requirements and is provided in the right format with the appropriate metadata information, it will be included in the database. Each time the database is updated through the iDiv Biodiversity portal (https://idata.idiv.de/), a new DOI will be generated for the whole updated database, ensuring the new contributors are acknowledged and become part of the ‘CESTES consortium’. This will allow storing the data on a permanent platform and prevent them from sinking into oblivion^30^.

### Structure of the database and the datasets

The 80 files currently in CESTES are structured into at least 8 sheets, depending on the original information and specificities of each dataset (Fig. 1).

The first four sheets include the processed core-data themselves:

1. “**comm**”: matrix of species abundances (68) or presences/absences (12), with species in columns and sites in rows (species are sometimes OTUs in some groups such as phytoplankton, or genus in some groups such as macroinvertebrates, or morphospecies where relevant).
2. “**traits**”: matrix of species trait information, i.e. any trait, be it functional, biological, life-history traits, either quantitative or categorical, functional group, etc., with traits in columns and species in rows.
3. “**envir**”: matrix of environmental variables in the broad sense of environment, i.e. any type of biotic and abiotic conditions or habitat characteristics relevant to the community of interest according to the original publication, with variables in columns and sites in rows.
4. “**coord**”: matrix of spatial coordinates, with X, the longitude and Y, the latitude as columns (in the Geographical Coordinate System as used in the original study) and sites in rows.

In every dataset, a “**DataKey**” sheet provides a description of all the entries of the four matrices (Fig. 4). Specific comments and information about any alteration applied to the dataset can be found in the “**Notes**” sheet, e.g. the species, or variables that were removed due to missing information, how the trait values were averaged across species when several measurements were available, how the original dataset was split into several datasets when there were several sampling periods, etc. The contact person for each dataset is also specified at the top of the “Notes” sheet of the dataset.

When the cleaning procedure implied changing the original datasets (see Data processing section above), we kept the information of the unaltered tables within the Excel file in separate sheets: “**commfull**”, “**traitsfull**”, “**coordfull**”, and/or “**envirfull**”.

The “**splist**” sheet includes the full list of taxa and the “**sitelist**” sheet, the list of sites. Both can provide additional information about the species (e.g. taxonomic classification) and the sites (e.g. regional information) when specified by the authors. Note that the species (site) names might not appear in the “splist” (“sitelist”) of all the datasets; this is because some authors preferred to provide their data in a redacted form, for instance, by censoring the species or the site names. As this does not hamper most of the analyses in community ecology, these datasets were integrated in the database.

Finally, when trait information was semi-quantitative and already fuzzy coded, we added a sheet “**blo**” to specify the Blocks information that is needed for weighting procedures in some trait analyses^31^.

### Description of the database

The CESTES database includes 80 datasets that cover different areas of the globe, ecosystem types, taxonomic groups, and spatial extents (Fig. 1). An overview of these datasets is presented in the Online-only Table 1.

### *ceste*, the non-spatial ancillary to CESTES

We provide access to 10 additional datasets that were not completely suitable for the CESTES database, due to the absence of spatial information or insufficient metadata but that were potentially valuable for their three other data matrices (see Online-only Table 3 attached to this manuscript). They follow the same structure as CESTES, except that they do not present the “coord” sheet and sometimes include only partial metadata. Some of the *ceste* datasets are likely to be enhanced in the near future and upgraded to the CESTES database as soon as they are made complete. *ceste* is stored in a zipped folder named “ceste.zip” that includes a series of 11 Excel files (10 data files + 1 metadata file) and can be found at the following links:

- Figshare: 10.6084/m9.figshare.c.4459637
-iDiv Biodiversity Portal: 10.25829/idiv.286-21-2695 (under the *Attachments* tab).

### CESTES, a live database

The current CESTES database is the starting point of a broader data-sharing project that aims to continue integrating new data as they become available, and as new contributors join the consortium by sharing their data.

In order to maintain the CESTES database in the future, we set up three measures to facilitate the data exchange and communication about the database:

1. a project website that advertises the database project and fosters data sharing: https://icestes.github.io/,
2. a tutorial to guide people on how to share their data (Supp. Mat. 1; https://icestes.github.io/sharedata),
3. a designated email address where people can send their data and ask questions about the CESTES project (cestes@idiv.de).

The data will be checked, curated, and integrated in the database through the iDiv Biodiversity Portal. This will update the database and generate a new DOI for the whole updated database, ensuring the new contributors are acknowledged.

### Citation of the individual datasets and of the database

Each CESTES and *ceste* dataset (CESTES^10,15,32–94^ and *ceste*^95–102^) is associated with reference(s) that should be cited in addition to the CESTES database *only if a single or few specific datasets are used separately from the database*. For instance, if one uses only Villéger’s datasets (Villeger2012a, b, c, d, and e), one would have to cite Villéger’s original study^10^ (to acknowledge the study antecedence), and the CESTES database^28^ (because it is through the CESTES database that the structured data and metadata were made available). The list of citations for each dataset is provided in Online-only Table 2. See also CESTES^29^ for updated versions of the live database and follow the last news about the database via https://icestes.github.io/posts/.

## Technical Validation

The technical validity of the CESTES database relies on five qualities pertaining to the datasets, and the overall database: the datasets (1) have individually been subject to peer-review process, (2) have reliable sampling properties, (3) have been thoroughly checked and cleaned, are ready-to-use for analyses and accompanied with metadata information; and the database (4) has a wide taxonomic and geographical coverage, and (5) will keep on extending in the future.

### Peer-reviewed data and TER relevance

All the datasets included in CESTES had already been the subject of publication(s) in peer reviewed scientific journals, or PhD theses (see Online-only Table 2). Therefore, each of the dataset has already received technical validation through both analysis and evaluation. In addition, since the focus of those studies was the species trait-environment relationships, the choice of the traits and environmental variables has already been the result of scientific reflection by the authors about the potential relevance of these variables with respect to the ecological context and the scale of study.

### Reliable sample properties

**Fig. 5:**
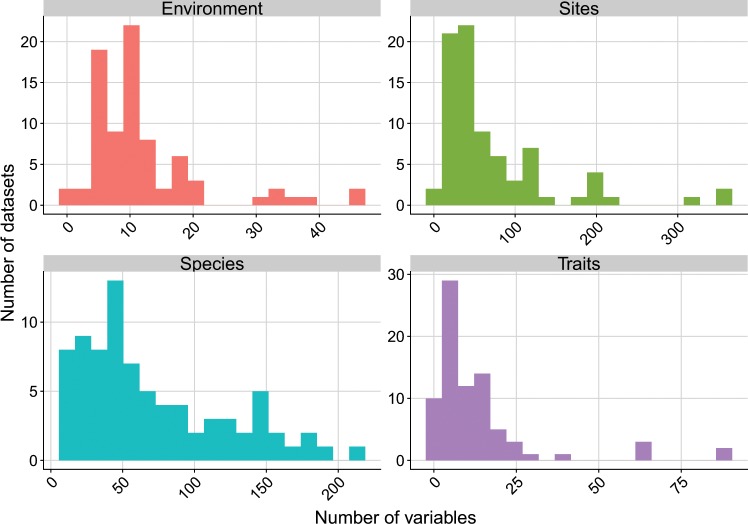
Data content of the CESTES database. Distribution of the number of environmental, site, species and trait variables across the datasets.

**Fig. 6:**
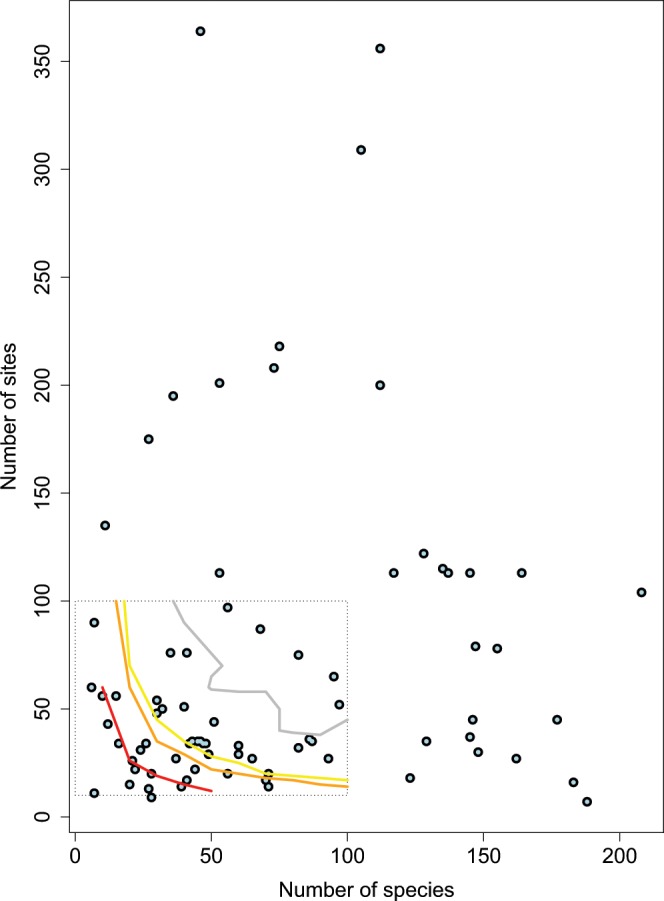
Power check of the CESTES datasets with respect to fourth-corner analysis’ requirements. After the Fig. 4a from Dray & Legendre^23^. Plot of the CESTES datasets (blue dots) according to their number of sites and number of species compared to three power thresholds observed in the fourth-corner simulation study from Dray & Legendre^23^. The dashed rectangle represents the range of values tested in the simulations of Dray & Legendre^23^. The curves represent their observed thresholds of Type II error rates - red = 30%, orange = 10%, yellow = 5%, grey = 0%. The datasets that fall below these thresholds are theoretically exposed to respectively 30%, 10%, 5% or 0% chance to fail to detect significant TERs with fourth-corner analysis although these exist. The figure shows that the majority of the CESTES datasets fall in a medium (70%) to very good (>95%) power zone (Power = 100% − Type II error).

The datasets include an average of 71 sites, 72 species, 12 environmental variables, and 14 traits (Fig. 5 & Online-only Table 1). In the particular context of fourth-corner analysis, Dray & Legendre^23^ showed that datasets with fewer than 30 species need to have substantially more than 30 sites in order for this multivariate method to perform well and detect existing TER. If we refer to the thresholds their simulation study found, we can say that 75% of the CESTES datasets can support multivariate analyses of a very good to good statistical power, i.e. have a Type II error risk of less than 10% (Fig. 6). The remaining 25% fall not far from the 30% limit, meaning that the risk of failing to detect significant TER although these exist is 30%. For these datasets, the users might need to be cautious if they intend to apply fourth-corner analysis and might need to consider other methods.

### Thorough data quality control and harmonized metadata information

Each dataset has been the subject of thorough manual checking for matching between site, species and traits number and identity across the four data matrices, the absence of empty sites, “ghost” species, NAs, and the consistency between the data received and the original publication. All dataset alterations that we applied are reported in the “Notes” sheet of the dataset file. Therefore, we offer a clean database while keeping full transparency on the steps taken to this end. A large effort was also invested in reporting precise and harmonized metadata information for every dataset (Fig. 4) so that CESTES users can easily have a full overview of the type of data.

### Wide taxonomic and geographical coverage

The geographical coverage of the database is global (Fig. 1) although as with most global compilations, there are clearly some regions of the world that are underrepresented, including Africa, Asia, India, Russia, which is typical^103^. To evaluate whether we might have missed potential datasets e.g., in Russia, we ran an additional literature search across a set of international journals specialised in Russian ecology (namely, Russian Journal of Ecology, Russian Journal of Marine Biology and Russian Journal of Biological Invasions). We used as search terms the simple association of “traits” AND “environment”. We found very few trait-based studies, only five studies that were relevant to the CESTES database, and only one that had spatial coordinates and agreed to share their data. In addition, we found a dearth of studies from North America. One reason for this was that a related database project focussing on plants primarily in the Americas has recently emerged^104^. Authors of plant datasets from these regions were less likely to respond to data request (only 17 out of 35 responded). This might also explain why, although our criteria covered all realms and taxonomic groups, we have a relatively low representation of plant data (i.e., less than 30% of our database, Fig. 2b) despite their large representation in other databases.

### Upgradeable and extendable database

Importantly, the CESTES database is upgradeable. Anyone interested to share data of this type and contribute is welcome to do so by contacting the corresponding author (AJ, cestes@idiv.de). We will maintain and add to the CESTES database in the future to enrich it with more taxa, ecosystem types, and locations in the world. On the longer term, three possible and very valuable extensions could include (1) individual-based traits measurements, since intraspecific variation in traits can be highly valuable to study environmental tracking^7,105,106^, (2) temporal measurements of both traits, environment, and species abundances in order to study the temporal variability of the TER^107^, (3) phylogenies corresponding to the recorded groups that would help draw inferences from community^108^ or macroecological patterns^8^. This would allow extending CESTES’ potential for synthesis work aiming to bridge metacommunity ecology, macroecology, and biodiversity-ecosystem functioning research.

## Usage Notes

In complement to the Excel version of CESTES, the database has also been stored as an.RData object to facilitate its further use for analyses in R^109^. This object is a list of lists. Each element of the first order list refers to one dataset, which itself is a list of four matrices; $comm, $envir, $traits and $coord.

First, this allows additional data processing. We set up R code routines (“CESTES_DataPrep.R”) that perform a thorough checking of the matrices, especially the match between the matrices’ dimensions, and the absence of empty sites, “ghost” species, and NAs.

The trait, environment, species, and coordinates variables were of mixed types (binary, categorical, ordinal, quantitative). To make the datasets properly readable and analysable by R, we made sure the numerical variables were treated as such by the program. We also re-coded the binary variables into 0/1 (numeric), the character and nominal variables into factors (this option can be turned off in the function), and, when relevant (i.e. when made explicit in the original publication), the numeric integer variables into ordinal variables (ordered factors).

Our R code routines generate data reports and send them to the working directory in the form of .txt files. These give the user different information on the “comm”, “traits”, “envir” and “coord” components of each dataset: list of variables and their types (factor, numeric, etc.), number of sites, species and traits, minimum and maximum value of the community data (that allows checking e.g., whether data are abundances or presences/absences).

The R code also applies some data transformation (e.g. scaling, Hellinger transformation, variable orthonormalization), generates spatial predictors (e.g. Moran Eigenvector Maps) and calculates some usual trait diversity metrics (e.g. Community Weighted Means, functional dispersion).

For all these processing, transformation and calculations, we used several R packages, available on CRAN, mainly *readxl*, *doBy*, *plyr*, *dplyr*, and *gdata* for the data processing, *vegan*, *ade4*, *stats* for the data transformation, *ape* and *adespatial* for the spatial processing, and *picante* and *FD* for the calculation of functional indices^109^.

All the R functions coded and used for the data preparation are provided in an R script “CESTES_DataPrep.R”. A fully processed and “ready-to-use” version of the CESTES database is stored as an .RData object called “CESTES.RData”.

Finally, further data plotting and metadata exploration are made possible via the R script “CESTES_Plots.R”, and the two metadata .csv files (“ListDat.csv”, “Metadat.csv”).

All these files (namely the database, the two R scripts, and the two metadata files) are stored in a zipped folder called “rCESTES.zip” in the “CESTES” folder at the following links:

- Figshare (fixed version): 10.6084/m9.figshare.c.4459637
- iDiv Biodiversity portal (evolutive version): 10.25829/idiv.286-21-2695 (under the *Primary Data* tab).

The flexibility of the iDiv Biodiversity Portal storage will allow us to keep updating, extending and sustaining the CESTES database and the R scripts in the future.

### Supplementary information

Supplementary File 1

## Data Availability

In addition to the Excel storage of the datasets, the CESTES database has also been stored as an.RData object to facilitate its further use for analyses in R^109^. It comes with R code scripts that allow further checking, processing, transforming and exploring the database content (for more details, see the Usage Notes section). We provide all this information in a folder called “rCESTES.zip” within the “CESTES” folder at the following links: Figshare repository (fixed version): 10.6084/m9.figshare.c.4459637. iDiv Biodiversity Portal (evolutive version): 10.25829/idiv.286-21-2695.

## Online-only Tables

**Online-only Table 1:** Overview of the CESTES database (name of the dataset, location, ecosystem type, spatial extent, number of sites, species, type of community data…).

DatasetName	Ecosystem and location	Study id	Taxonomic group	Ecosystem type	Extent (km2)	Level of human disturbance	Sampling date(s)/period	nbEnv	nbTra	nbSpe	nbSit	Example of traits	Example of environmental variables	Type of community data	References
Bagaria2012	Mediterranean semi-natural mountain grasslands, southern Catalonia, Spain	1	Plants	Terrestrial	2000	Semi-natural	2007	8	13	49	29	Seed size|Dispersal type|Corolla type|Flower/pseudanthium size|Resprouting ability after fire	Mean annual temperature|Mean annual precipitation|Past patch area|Current patch area|% of patch area reduction	number of individuals	Bagaria, G., Pino, J., Rodà, F., & Guardiola, M. (2012). Species traits weakly involved in plant responses to landscape properties in Mediterranean grasslands. Journal of Vegetation Science, 23(3), 432–442. doi:10.1111/j.1654-1103.2011.01363.x
Barbaro2009a	Intensive pine plantations, mosaic forest landscapes in south-western France	2	Beetles	Terrestrial	32.16	Forestry	2002–2003	11	12	36	195	European trend|European rarity|Regional rarity|Biogeographical position|Daily activity	Clearcut cover/Crop cover|Edge density|Mean patch area|Schrub land cover/ Clearcut cover/Crop cover|Shannon diversity index	number of individuals	Barbaro, L., & van Halder, I. (2009). Linking bird, carabid beetle and butterfly life‐history traits to habitat fragmentation in mosaic landscapes. Ecography, 32(2), 321–333. doi:10.1111/j.1600-0587.2008.05546.x
Barbaro2009b	Intensive pine plantations, mosaic forest landscapes in south-western France	2	Birds	Terrestrial	32.16	Forestry	2002–2003	11	12	53	201	National trend|Foraging technique|Diet|Nest location|Clutch size	Clearcut cover/Crop cover|Edge density|Mean patch area|Schrub land cover/ Clearcut cover/Crop cover|Shannon diversity index	abundance index	Barbaro, L., & van Halder, I. (2009). Linking bird, carabid beetle and butterfly life‐history traits to habitat fragmentation in mosaic landscapes. Ecography, 32(2), 321–333. doi:10.1111/j.1600-0587.2008.05546.x
Barbaro2012	Fragmented native forests, volcanic banks peninsula, Canterbury, South Island, New Zealand	3	Birds	Terrestrial	625	Natural	2010–2011	6	7	21	26	Biogeographic origin|Foraging method|Body mass|Adult diet|Mobility	Plot location|Plot elevation|Patch area size|Forest percentage|Grassland precentage	number of individuals	Barbaro, L., Brockerhoff, E. G., Giffard, B., & van Halder, I. (2012). Edge and area effects on avian assemblages and insectivory in fragmented native forests. Landscape Ecology, 27(10), 1451–1463. doi:10.1007/s10980-012-9800-x
Barbaro2017	Vineyards, Aquitaine, France	4	Birds	Terrestrial	750	Agricultural	2013	6	8	56	20	Diet in a breeding season|Foraging guild|Clutch size|Body mass|Nesting site	Grass cover|SNH in 100 meters buffer|SNH in 250 meters buffer|SNH in 500 meters buffer|SNH in 750 meters buffer	number of individuals	Barbaro, L., Rusch, A., Muiruri, E. W., Gravellier, B., Thiery, D., & Castagneyrol, B. (2017). Avian pest control in vineyards is driven by interactions between bird functional diversity and landscape heterogeneity. Journal of Applied Ecology, 54(2), 500–508. doi:10.1111/1365-2664.12740
Bartonova2016	National Nature Reserves and National Natural Monuments, Czech Republic	5	Butterflies	Terrestrial	78866	Natural	2004–2006	11	13	128	122	body size|mobility|density|voltinism|flight period lenght	Relative Perimeter (Perimeter/Area)|Average altitude [m a.s.l.]|range of altitudes [m]|prevailing biotope type|Reserve area [m2]	abundance class	Bartonova, A., Benes, J., Fric, Z. F., Chobot, K., & Konvicka, M. (2016). How universal are reserve design rules? A test using butterflies and their life history traits. Ecography, 39(5), 456–464. doi:10.1111/ecog.01642
Bonada2007S	Mediterranean rivers, Catalonia, Spain	6	Macroinvertebrates	Freshwater	96.3	Natural	summer 1996	16	63	70	17	Maximal size|Life cycle duration|Potential number of reproduction cycles per year|Aquatic stages|Reproduction	Discharge (l/s)|Temperature (ºC)|Conductivity (microS/cm)|pH|Oxygen (mg/l)	average number of individuals	Bonada, N., Rieradevall, M., & Prat, N. (2007). Macroinvertebrate community structure and biological traits related to flow permanence in a Mediterranean river network. Hydrobiologia, 589(1), 91–106. doi:10.1007/s10750-007-0723-5
Bonada2007W	Mediterranean rivers, Catalonia, Spain	6	Macroinvertebrates	Freshwater	96.3	Natural	winter 1996	14	63	44	22	Maximal size|Life cycle duration|Potential number of reproduction cycles per year|Aquatic stages|Reproduction	Discharge (l/s)|Temperature (ºC)|Conductivity (microS/cm)|pH|Oxygen (mg/l)	average number of individuals	Bonada, N., Rieradevall, M., & Prat, N. (2007). Macroinvertebrate community structure and biological traits related to flow permanence in a Mediterranean river network. Hydrobiologia, 589(1), 91–106. doi:10.1007/s10750-007-0723-5
BrindAmour2011a	Drouin lake, Laurentian Shield Lakes, Quebec, Canada	7	Fishes	Freshwater	0.31	Semi-natural	2001	19	24	7	90	Type of diet|Feeding strata|Body morphology|Migration|Mouth position	Mean littoral slope|Riparian slope|Mean depth|Substrate: Sand|Substrate: Rock	abundance class	Brind’Amour, A., Boisclair, D., Dray, S., & Legendre, P. (2011). Relationships between species feeding traits and environmental conditions in fish communities: a three-matrix approach. Ecological Applications, 21(2), 363–377. doi:10.1890/09-2178.1
BrindAmour2011b	Pare lake, Laurentian Shield Lakes, Quebec, Canada	7	Fishes	Freshwater	0.23	Semi-natural	2001	17	24	6	60	Type of diet|Feeding strata|Body morphology|Migration|Mouth position	Mean littoral slope|Riparian slope|Mean depth|Substrate: Sand|Substrate: Rock	abundance class	Brind’Amour, A., Boisclair, D., Dray, S., & Legendre, P. (2011). Relationships between species feeding traits and environmental conditions in fish communities: a three-matrix approach. Ecological Applications, 21(2), 363–377. doi:10.1890/09-2178.1
Campos2018	Tropical floodplain lakes, Upper Paraná River floodplain, Brazil	8	Ostracods	Freshwater	700	Mixed	2011	7	2	37	27	Swimming behavior|Body size	Water temperature|Acidity|Electrical condutivity|Dissolved oxygen|Lake perimeter	density	Campos, R. de, Lansac-Tôha, F. M., Conceição, E. de O. da, Martens, K., & Higuti, J. (2018). Factors affecting the metacommunity structure of periphytic ostracods (Crustacea, Ostracoda): a deconstruction approach based on biological traits. Aquatic Sciences, 80(2), 16. doi:10.1007/s00027-018-0567-2
Carvalho2015	Tocantins-Araguaia river basin, Amazonia, Brazil	9	Stream fishes	Freshwater	180000	Mixed	2008	8	26	65	27	BodyMass_grams|Trophic Guild|Parental Care|Water Column Position|Foragin Method	Altitude (m)|Channel_Depth (m)|Channel_Width (m)|Turbidity (NTU)|Dissolved Oxygen	presence/absence	Carvalho, R. A., & Tejerina-Garro, F. L. (2015). The influence of environmental variables on the functional structure of headwater stream fish assemblages: a study of two tropical basins in Central Brazil. Neotropical Ichthyology, 13(2), 349–360. doi:10.1590/1982-0224-20130148
Castro2010	Southern Portugal	10	Plants	Terrestrial	1.9844	Agricultural	NA	8	6	28	9	Surface leaf area|Leaf dry matter content|Leaf nitrogen content|Leaf carbon content|Leaf phosphorus content	Soil nitrogen contect|Soil carbon content|Soil phosphorous contect|Organic matter in soil|Soil Carbon	percentage cover	Castro, H., Lehsten, V., Lavorel, S., & Freitas, H. (2010). Functional response traits in relation to land use change in the Montado. Agriculture, Ecosystems & Environment, 137(1–2), 183–191. doi:10.1016/j.agee.2010.02.002
Charbonnier2016a	Forests, Europe	11	Bats	Terrestrial	4400000	Forestry	2012–2013	5	9	27	175	Foraging behaviours|Diet type|Nest or roost site location|Migration status|Breeding date	Plot altitude|Forest compositoin|Mean temperature|Mean precipitation|Deciduous cover	number of individuals	Charbonnier, Y. M., Barbaro, L., Barnagaud, J.-Y., Ampoorter, E., Nezan, J., Verheyen, K., & Jactel, H. (2016). Bat and bird diversity along independent gradients of latitude and tree composition in European forests. Oecologia, 182(2), 529–537. doi:10.1007/s00442-016-3671-9
Charbonnier2016b	Forests, Europe	11	Birds	Terrestrial	4400000	Forestry	2012–2013	5	10	73	208	Foraging behaviours|Diet type|Nest or roost site location|Migration status|Breeding date	Plot altitude|Forest compositoin|Mean temperature|Mean precipitation|Deciduous cover	number of individuals	Charbonnier, Y. M., Barbaro, L., Barnagaud, J.-Y., Ampoorter, E., Nezan, J., Verheyen, K., & Jactel, H. (2016). Bat and bird diversity along independent gradients of latitude and tree composition in European forests. Oecologia, 182(2), 529–537. doi:10.1007/s00442-016-3671-9
Chmura2016	Karkonosze Mts, Sudeten Mts, Poland	12	Plants	Terrestrial	135.05	Natural	NA	10	17	46	364	Species Leaf Area (mean value)|Plant height (mean value)|Seed mass|Seed dispersal type|Rosette (leaf arrangement)	Bryophytes cover|Decomposition stage|Length of a log |Area of a log surface|Moisture of a lo	number of individuals	Chmura, D., Żarnowiec, J., & Staniaszek-Kik, M. (2016). Interactions between plant traits and environmental factors within and among montane forest belts: A study of vascular species colonising decaying logs. Forest Ecology and Management, 379, 216–225. doi:10.1016/j.foreco.2016.08.024
Choler2005	Southwestern Alps, Aravo, Grand Galibier, France	55	Plants	Terrestrial	0.02	Semi-natural	2001	7	8	82	75	vegetative height|lateral spread|leaf elevation angle|Specific leaf area|leaf nitrogen	relative south aspects|slope|microtopographic landform index|physical disturbance|zoogenic disturbance	classes of percentage cover	Choler, P. (2005). Consistent Shifts in Alpine Plant Traits along a Mesotopographical Gradient. Arctic, Antarctic, and Alpine Research, 37(4), 444–453. Retrieved from http://www.jstor.org/stable/4095863
ChongSeng2012a	Seychelles archipelago	13	Coral reef fishes	Marine	3600	Semi-natural	2010	17	2	147	79	Fish functional group|Fishing pressure	Acropora coral|Crustose coralline algae|Leathery macroalgae|Non biological particles|Other benthic organisms	number of individuals	Chong-Seng, K. M., Mannering, T. D., Pratchett, M. S., Bellwood, D. R., & Graham, N. A. J. (2012). The Influence of Coral Reef Benthic Condition on Associated Fish Assemblages. PLOS ONE, 7(8), e42167. doi:10.1371/journal.pone.0042167
ChongSeng2012b	Seychelles archipelago	13	Coral reef fishes	Marine	3600	Semi-natural	2012	12	2	155	78	Fish functional group|Fishing pressure	Acropora coral|Crustose coralline algae|Leathery macroalgae|Non biological particles|Other benthic organisms	number of individuals	Chong-Seng, K. M., Mannering, T. D., Pratchett, M. S., Bellwood, D. R., & Graham, N. A. J. (2012). The Influence of Coral Reef Benthic Condition on Associated Fish Assemblages. PLOS ONE, 7(8), e42167. doi:10.1371/journal.pone.0042167
Cleary2007a	Mentaya river, Central Kalimantan province, Borneo, Indonesia	14	Birds	Terrestrial	196	Mixed	1997–1998	36	4	145	37	Feeding Guild|Global distribution|Size|Conservation status	Logging|Slope position|Elevation|Short saplings|Tall saplings	log transformed number of individuals	Cleary, Daniel F. R., Boyle, T. J. B., Setyawati, T., Anggraeni, C. D., Loon, E. E. V., & Menken, S. B. J. (2007). Bird species and traits associated with logged and unlogged forest in Borneo. Ecological Applications, 17(4), 1184–1197. doi:10.1890/05-0878
Cleary2007b	Coral reefs, Spermonde Archipelago, Makassar, southwest Sulawesi, Indonesia	15	Foraminifera	Marine	2418	Mixed	1997	10	3	24	31	Species form|Symbiont-bearing foraminifera|Skeletal structure	Maximal sea depth|Maximal distance|Exposure to oceanic currents|Sedimentation|Coral formation	number of individuals	Cleary, Daniel F. R., & Renema, W. (2007). Relating species traits of foraminifera to environmental variables in the Spermonde Archipelago, Indonesia. MARINE ECOLOGY PROGRESS SERIES, 334, 73–82. doi:10.3354/meps334073
Cleary2016	Coral reefs, Jakarta, Indonesia	16	Fishes	Marine	1764	Fishing	2005	21	15	162	27	Trophic level?|Life expectancy?|Age maturity?	Water transparency|Temperature|Acidity|Dissolved oxygen|Macroalgae	number of individuals	Cleary, D. F. R., Polónia, A. R. M., Renema, W., Hoeksema, B. W., Rachello-Dolmen, P. G., Moolenbeek, R. G., … de Voogd, N. J. (2016). Variation in the composition of corals, fishes, sponges, echinoderms, ascidians, molluscs, foraminifera and macroalgae across a pronounced in-to-offshore environmental gradient in the Jakarta Bay–Thousand Islands coral reef complex. Marine Pollution Bulletin, 110(2), 701–717. doi:10.1016/j.marpolbul.2016.04.042
Cornwell2009	Jasper Ridge Biological Preserve, Coastal, California, USA	17	Woody plants	Terrestrial	4.81	Semi-natural	2002–2003	3	3	42	34	geometric mean of specific leaf area measurements|arithmetic mean of specific leaf area measurments|number of samples in the species meanpotential diurnal insolation	elevation above sea level|soil water content	percentage cover	Cornwell, W. K., & Ackerly, D. D. (2009). Community Assembly and Shifts in Plant Trait Distributions across an Environmental Gradient in Coastal California. Ecological Monographs, 79(1), 109–126. doi: 10.1890/07-1134.1
Diaz2008	Segura River basin,SE Spain	18	Macroinvertebrates	Freshwater	6300	Mixed	1999–2001	39	62	208	104	Maximal size|Life cycle duration|Potential No. reproductive cycles per year|Aquatic stages|Reproduction	sampling date (month year)|total suspended solids|Ammonium|Nitrite|Nitrate	number of individuals	Mellado-Diaz, A., Luisa Suarez Alonso, M., & Rosario Vidal-Abarca Gutierrez, M. (2008). Biological traits of stream macroinvertebrates from a semi-arid catchment: patterns along complex environmental gradients. FRESHWATER BIOLOGY, 53(1), 1–21. doi:10.1111/j.1365-2427.2007.01854.x
Doledec1996	Urban-rural gradient, Lyon, France	19	Birds	Terrestrial	96	Mixed	1981	11	4	40	51	Feeding habit|Feeding stratum|Breeding stratum|Migratory strategy	Presence of farms or villages|Presence of small buildings|Presence of high buildings|Presence of industry|Presence of fields	abundance class	Dolédec, S., Chessel, D., Braak, C. J. F. ter, & Champely, S. (1996). Matching species traits to environmental variables: a new three-table ordination method. Environmental and Ecological Statistics, 3(2), 143–166. doi:10.1007/BF02427859
Drew2017	Archipelagos, Melanesia	20	Coral reef fishes	Marine	15300000	Mixed	NA	1	3	188	7	Schooling behavior|Maximal body size|Larvae development duration	Reef Area	presence/absence	Drew, J. A., & Amatangelo, K. L. (2017). Community assembly of coral reef fishes along the Melanesian biodiversity gradient. PLoS ONE, 12(10). doi:10.1371/journal.pone.0186123
Dziock2011	Dessau, Magdeburg, Elbe, Floodplain, Sachsen-Anhalt, Germany	21	Grasshopers	Terrestrial	224	Agricultural	2006	5	6	16	34	Dispersal ability|Passive dispersal ability|Ovarioles number|Body size|Oviposition in plant material	Elevation|Distance to the river|Class of litter cover|Vegetation height|Land use and intensity	abundance class	Dziock, F., Gerisch, M., Siegert, M., Hering, I., Scholz, M., & Ernst, R. (2011). Reproducing or dispersing? Using trait based habitat templet models to analyse Orthoptera response to flooding and land use. Agriculture, Ecosystems & Environment, 145(1), 85–94. doi:10.1016/j.agee.2011.07.015 Klaiber, J., Altermatt, F., Birrer, S., Chittaro, Y., Dziock, F., Gonseth, Y., … Bergamini, A. (2017). Fauna Indicativa (Report). Eidg. Forschungsanstalt für Wald, Schnee und Landschaft WSL, CH-Birmensdorf. Retrieved from http://orgprints.org/34497/
Eallonardo2013	Inland salt/marsh, New York State, USA, near Montezuma; Carncross, Howland Island and Fox Ridge	54	Plants	Mixed	3.5	Natural	2007	14	14	41	76	Perennial life span|Rhizomatous growth|Gramonoid growth form|C4 photosynthetic pathway|Succulence	Electrical conductivity|extractible cation concentration|pH|total nitrogen|flooding duration	relative percentage cover	Eallonardo, A. S., Leopold, D. J., Fridley, J. D., & Stella, J. C. (2013). Salinity tolerance and the decoupling of resource axis plant traits. Journal of Vegetation Science, 24(2), 365–374. doi:10.1111/j.1654-1103.2012.01470.x
Farneda2015	Biological Dynamics of Forest Fragments Project (BDFFP) located ca. 80 km north of Manaus, Central Amazon, Brazil	22	Bats	Terrestrial	680	Natural	2011–2013	9	8	41	17	trophic_level|habitat_classification|body_mass|dietary_specialization|vertical_stratification	Average number of trees|Average tree diameter|Average vertical stratification|Average number of lianas|Average number of potential roosts	average number of individuals	Farneda, F. Z., Rocha, R., López-Baucells, A., Groenenberg, M., Silva, I., Palmeirim, J. M., … Meyer, C. F. J. (2015). Trait-related responses to habitat fragmentation in Amazonian bats. Journal of Applied Ecology, 52(5), 1381–1391. doi:10.1111/1365-2664.12490
Frenette2012a	Arid steppes, Eastern Morocco	23	Plants	Terrestrial	11765	Mixed	2009	5	18	32	50	Leaf Area|Specific Leaf Area|Leaf Dry Matter Content|Leaf Carbon 13 Isotope Content|Leaf Nitrogen 15 Isotope Content	name of the regional site|Grazing|Aridity index |Duration of exclosures|Elevation	number of individuals	Frenette-Dussault, C., Shipley, B., Léger, J.-F., Meziane, D., & Hingrat, Y. (2012). Functional structure of an arid steppe plant community reveals similarities with Grime’s C-S-R theory. Journal of Vegetation Science, 23(2), 208–222. doi:10.1111/j.1654-1103.2011.01350.x
Frenette2012b	Arid steppes, Eastern Morocco	23	Plants	Terrestrial	11765	Mixed	2010	5	18	32	50	Leaf Area|Specific Leaf Area|Leaf Dry Matter Content|Leaf Carbon 13 Isotope Content|Leaf Nitrogen 15 Isotope Content	name of the regional site|Grazing|Aridity index |Duration of exclosures|Elevation	number of individuals	Frenette-Dussault, C., Shipley, B., Léger, J.-F., Meziane, D., & Hingrat, Y. (2012). Functional structure of an arid steppe plant community reveals similarities with Grime’s C-S-R theory. Journal of Vegetation Science, 23(2), 208–222. doi:10.1111/j.1654-1103.2011.01350.x
Frenette2013	Arid steppes, Eastern Morocco	23	Ants	Terrestrial	11765	Mixed	2010	5	6	22	22	Feeding|Period of activity|Recoded period of activity|Color|Functional group	Site region|Grazing|Aridity index|Duration of exclosures|Elevation	number of individuals	Frenette-Dussault, C., Shipley, B., & Hingrat, Y. (2013). Linking plant and insect traits to understand multitrophic community structure in arid steppes. Functional Ecology, 27(3), 786–792. doi:10.1111/1365–2435.12075
Fried2012	Agriculture areas, France	24	Plants	Terrestrial	386000	Agricultural	2003–2006	11	10	75	218	Plant life form|Mode of species dispersal|Plant class|Plant height|Seed weight	Temperature|Preciptiation|Soil pH|Sowing date|Tillage depth	abundance class	Fried, G., Kazakou, E., & Gaba, S. (2012). Trajectories of weed communities explained by traits associated with species’ response to management practices. Agriculture, Ecosystems & Environment, 158, 147–155. doi:10.1016/j.agee.2012.06.005
Gallardo2009	Ebro river, Mediterranee, Spain	25	Macroinvertebrates	Freshwater	11	Agricultural	2006	30	87	35	76	Maximal size|Respiration|Life cycle duration|Potential number of reproduction cycles per year|Reproduction	Type of wetland|position in the watershed|sampling season|emergent vegetation|submerged vegetation	number of individuals	Gallardo, B., Gascon, S., Garcia, M., & Comin, F. A. (2009). Testing the response of macroinvertebrate functional structure and biodiversity to flooding and confinement. Journal of Limnology, 68(2), 315–326. doi: 10.3274/JL09-68-2-14
Gibb2015	Themeda grasslands, south-east Australia	26	Spiders	Terrestrial	37.64970119	Mixed	2009–2011	7	10	86	36	Sex|Body length|Abdomen length|Abdomen width|Cephalothorax widt	Elevation (m)|Temperature (ºC)|Precipitation (mm)|Grass height|Disturbance	number of individuals	Gibb, H., Muscat, D., Binns, M. R., Silvey, C. J., Peters, R. A., Warton, D. I., & Andrew, N. R. (2015). Responses of foliage-living spider assemblage composition and traits to a climatic gradient in Themeda grasslands: Spider Traits and Climatic Gradients. Austral Ecology, 40(3), 225–237. doi:10.1111/aec.12195
Goncalves2010	Santa Lucia Biological Station (SLBS), Santa Teresa County, Espirito Santo State, southeast Brazil	27	Spiders	Terrestrial	0.44	Natural	2006–2007	1	4	146	45	Prosoma height|Prosoma width|Prosoma length|Opistosoma length	Habitat type	number of individuals	Gonçalves-Souza, T., Brescovit, A. D., de C. Rossa-Feres, D., & Romero, G. Q. (2010). Bromeliads as biodiversity amplifiers and habitat segregation of spider communities in a Neotropical rainforest. The Journal of Arachnology, 38(2), 270–279. Retrieved from https://www.jstor.org/stable/20788618
Goncalves2014a	Open restingas, Atlantic rainforest, Brazil	28	Spiders	Terrestrial	220000	Natural	2009	10	22	105	309	Guild|Prosoma height|Prosoma width|Prosoma length|Opistosoma length	Plant species|Crown height|Higher crown length|Lower crown length|Leaf length	number of individuals	Gonçalves-Souza, T., Brescovit, A. D., de C. Rossa-Feres, D., & Romero, G. Q. (2010). Bromeliads as biodiversity amplifiers and habitat segregation of spider communities in a Neotropical rainforest. The Journal of Arachnology, 38(2), 270–279. Retrieved from https://www.jstor.org/stable/20788618
Goncalves2014b	Open restingas, Atlantic rainforest, Brazil	28	Spiders	Terrestrial	220000	Natural	2010	10	22	112	356	Guild|Prosoma height|Prosoma width|Prosoma length|Opistosoma length	Plant species|Crown height|Higher crown length|Lower crown length|Leaf length	number of individuals	Gonçalves-Souza, T., Romero, G. Q., & Cottenie, K. (2014). Metacommunity versus Biogeography: A Case Study of Two Groups of Neotropical Vegetation-Dwelling Arthropods. PLOS ONE, 9(12), e115137. doi:10.1371/journal.pone.0115137
Jamil2013	Terschelling island, dune meadow, Netherlands	29	Plants	Terrestrial	84	Agricultural	1982	5	5	28	20	Specific Leaf Area|Canopy height of a shoot|Lead dry matter content|Seed mass|Life span	A1 horizon thickness|Moisture|Grassland management type|Use|Manure	abundance class	Jamil, T., Ozinga, W. A., Kleyer, M., & ter Braak, C. J. F. (2013). Selecting traits that explain species-environment relationships: a generalized linear mixed model approach. Journal of Vegetation Science, 24(6), 988–1000. doi:10.1111/j.1654-1103.2012.12036.x
Jeliazkov2013	Ponds, agricultural areas, Brie, Seine-et-Marne, France	30	Macroinvertebrates	Freshwater	430	Agricultural	2012	47	91	112	200	Body size class|Body size class|Body size class|Body size class|Body size class	Pond habitat and context|Pond area|Agricultural gradient|Urban gradient|Fish or amphibian presence	number of individuals	Jeliazkov, A. (2013). Scale-effects in agriculture-environment-biodiversity relationships (Doctoral thesis). Université Pierre et Marie Curie, Paris, France. Retrieved from http://www.sudoc.fr/180446460
Jeliazkov2014	Ponds, agricultural areas, Brie, Seine-et-Marne, France	30	Amphibians	Freshwater	430	Agricultural	2011–2012	9	16	11	135	Vertical foraging stratum: fossorial|Vertical foraging stratum: terrestrial|Vertical foraging stratum: aquatic|Vertical foraging stratum: arboreal|Diet: Arthropods	Fish presence|Water Quality Index|Pond habitat and context|Proportion of wooded habitat|Pond density	number of individuals	Jeliazkov, A., Chiron, F., Garnier, J., Besnard, A., Silvestre, M., & Jiguet, F. (2014). Level-dependence of the relationships between amphibian biodiversity and environment in pond systems within an intensive agricultural landscape. Hydrobiologia, 723(1), 7–23. doi:10.1007/s10750-013-1503-z
Krasnov2015	Palearctic area; Slovakia	31	Flea	Terrestrial	33000000	Mixed	1958, 2008	17	13	177	45	Abundance|Host number|Number of host exploted across region|Number of host exploted across continent|Host number correlaiton	Size of the area|Mean altitude|Minimal altitude|Maximal altitude|NDVI for autumn	presence/absence	Krasnov, B. R., Shenbrot, G. I., Khokhlova, I. S., Stanko, M., Morand, S., & Mouillot, D. (2015). Assembly rules of ectoparasite communities across scales: combining patterns of abiotic factors, host composition, geographic space, phylogeny and traits. Ecography, 38(2), 184–197. doi:10.1111/ecog.00915
Lowe2018a	Urban gradient, Sydney, Australia	32	Spiders	Terrestrial	1000	Mixed	2013	33	7	135	115	Guild|Hunting style|Capture lines|Body Size|Period of activity	Land use type|Microhabitat (0–50 cm)|Microhabitat (50–100 cm)|Microhabitat (100–200 cm)|Leaf litter in microhabitat	number of individuals	Lowe, E. C., Threlfall, C. G., Wilder, S. M., & Hochuli, D. F. (2018). Environmental drivers of spider community composition at multiple scales along an urban gradient. Biodiversity and Conservation, 27(4), 829–852. doi:10.1007/s10531-017-1466-x
Lowe2018b	Urban gradient, focus on gardens, Sydney, Australia	32	Spiders	Terrestrial	1000	Mixed	2013	20	7	95	65	Guild|Hunting style|Capture lines|Body Size|Period of activity	Site region|Stoires number|Adjoining backyards|Distance between the bushes|Distance between fragments	number of individuals	Lowe, E. C., Threlfall, C. G., Wilder, S. M., & Hochuli, D. F. (2018). Environmental drivers of spider community composition at multiple scales along an urban gradient. Biodiversity and Conservation, 27(4), 829–852. doi:10.1007/s10531-017-1466-x
Marteinsdottir2014	Grazed ex-arable fields and semi-natural grasslands, southeast Sweden	33	Plants	Terrestrial	12	Mixed	2007–2008	7	3	39	14	Specific leaf area|Leaf drymatter content|Seed mass	pH|Ammonium|Phosphorus|Moisture|Potassium	average percentage cover	Marteinsdóttir, B., & Eriksson, O. (2014). Plant community assembly in semi-natural grasslands and ex-arable fields: a trait-based approach. Journal of Vegetation Science, 25(1), 77–87. doi:10.1111/jvs.12058
Meffert2013	Urban wasteland, Berlin, Germany	34	Birds	Terrestrial	892	Urban	2007	4	5	30	54	Food type|Foraging technique|Adult survival|Innovation rate|Migration strategy	Population within 50 m|Population within 200 m|Sealing within 50 m|Sealing within 2000 m	density	Meffert, P. J., & Dziock, F. (2013). The influence of urbanisation on diversity and trait composition of birds. Landscape Ecology, 28(5), 943–957. doi:10.1007/s10980-013-9867-z
Ossola2015	Urban habitat, south-eastern Melbourne, Australia	35	Ants	Terrestrial	100	Urban	2013–2014	20	5	60	29	Head width|Head lendth|Femur length|Pronotum width|Body size index	Habitat type |Understory volume total|Soil cover|Litter cover|Litter mass	number of individuals	Ossola, A., Nash, M. A., Christie, F. J., Hahs, A. K., & Livesley, S. J. (2015). Urban habitat complexity affects species richness but not environmental filtering of morphologically-diverse ants. PeerJ, 3, e1356. doi:10.7717/peerj.1356
Pakeman2011	Drumbuie, Scotland	36	Plants	Terrestrial	35	Agricultural	2007	33	28	148	30	Flowering start, month|Flowering end, month|log(seed mass)|variance in seed dimensions|leafing period, summer green	SoilN|SoilC|Soil_C:N|MoistureLoss|LossOnIgnition	relative abundances	Pakeman, R. J. (2011). Multivariate identification of plant functional response and effect traits in an agricultural landscape. Ecology, 92(6), 1353–1365. doi:10.1890/10-1728.1
Pavoine2011	Coastal marsh plain Mekhada in the east of Annaba, La Mafragh, Algeria	37	Plants	Terrestrial	100	Agricultural	1979	8	14	56	97	Anemogamous|Autogamous|Entomogamous|Annual|Biennial	Clay|Silt|Sand|K2O|Mg2+	number of individuals	Pavoine, S., Vela, E., Gachet, S., de Bélair, G., & Bonsall, M. B. (2011). Linking patterns in phylogeny, traits, abiotic variables and space: a novel approach to linking environmental filtering and plant community assembly: Multiple data in community organization. Journal of Ecology, 99(1), 165–175. doi:10.1111/j.1365-2745.2010.01743.x
Pekin2011	Walpole and Albany, SW Australia	38	Plants	Terrestrial	1073	Semi-natural	2007	17	4	183	16	Life cycle|Regeneration strategy|Root structure|N-fixing ability	Soil type|Fire interval sequence|Fire frequency|Mean annual precip.|Potential evapotrans.	number of individuals	Pekin, B. K., Wittkuhn, R. S., Boer, M. M., Macfarlane, C., & Grierson, P. F. (2011). Plant functional traits along environmental gradients in seasonally dry and fire-prone ecosystem. Journal of Vegetation Science, 22(6), 1009–1020. doi: 10.1111/j.1654-1103.2011.01323.x
Pomati2013	peri-alpine mesotrophic Lake Zürich, Switzerland	39	Phytoplankton	Freshwater	88.66	Mixed	2009	8	15	20	15	Phytoplankton morphology|Phytoplankton fluorescence|Length by SWS|Total fluorescence, yellow|Total fluorescence, orange	Water depth|Water temperature|Water conductivity|Oxygen level|Dissolved organic carbon	concentration	Pomati, F., Kraft, N. J. B., Posch, T., Eugster, B., Jokela, J., & Ibelings, B. W. (2013). Individual Cell Based Traits Obtained by Scanning Flow-Cytometry Show Selection by Biotic and Abiotic Environmental Factors during a Phytoplankton Spring Bloom. PLoS ONE, 8(8), e71677. doi:10.1371/journal.pone.0071677
Purschke2012a	Semi-natural grasslands, Jordtorp area, Öland Baltic Island, Sweden	40	Plants	Terrestrial	20.25	Semi-natural	2007	12	2	164	113	Adult plant longevity|epizoochorous dispersal potential	percentage of grassland habitat in 1994|percentage of grassland habitat in 1938|percentage of grassland habitat in 1835|diversity of the landscape matrix in 1994|diversity of the landscape matrix in 1938	presence/absence	Purschke, O., Sykes, M. T., Reitalu, T., Poschlod, P., & Prentice, H. C. (2012). Linking landscape history and dispersal traits in grassland plant communities. Oecologia, 168(3), 773–783. doi:10.1007/s00442-011-2142-6
Purschke2012b	Semi-natural grasslands, Jordtorp area, Öland Baltic Island, Sweden	40	Plants	Terrestrial	20.25	Semi-natural	2007	12	1	53	113	Endozoochorous dispersal potential	percentage of grassland habitat in 1994|percentage of grassland habitat in 1938|percentage of grassland habitat in 1835|diversity of the landscape matrix in 1994|diversity of the landscape matrix in 1938	presence/absence	Purschke, O., Sykes, M. T., Reitalu, T., Poschlod, P., & Prentice, H. C. (2012). Linking landscape history and dispersal traits in grassland plant communities. Oecologia, 168(3), 773–783. doi:10.1007/s00442-011-2142-6
Purschke2012c	Semi-natural grasslands, Jordtorp area, Öland Baltic Island, Sweden	40	Plants	Terrestrial	20.25	Semi-natural	2007	12	1	145	113	Wind dispersal potential	percentage of grassland habitat in 1994|percentage of grassland habitat in 1938|percentage of grassland habitat in 1835|diversity of the landscape matrix in 1994|diversity of the landscape matrix in 1938	presence/absence	Purschke, O., Sykes, M. T., Reitalu, T., Poschlod, P., & Prentice, H. C. (2012). Linking landscape history and dispersal traits in grassland plant communities. Oecologia, 168(3), 773–783. doi:10.1007/s00442-011-2142-6
Purschke2012d	Semi-natural grasslands, Jordtorp area, Öland Baltic Island, Sweden	40	Plants	Terrestrial	20.25	Semi-natural	2007	12	1	117	113	Seed longevity index	percentage of grassland habitat in 1994|percentage of grassland habitat in 1938|percentage of grassland habitat in 1835|diversity of the landscape matrix in 1994|diversity of the landscape matrix in 1938	presence/absence	Purschke, O., Sykes, M. T., Reitalu, T., Poschlod, P., & Prentice, H. C. (2012). Linking landscape history and dispersal traits in grassland plant communities. Oecologia, 168(3), 773–783. doi:10.1007/s00442-011-2142-6
Purschke2012e	Semi-natural grasslands, Jordtorp area, Öland Baltic Island, Sweden	40	Plants	Terrestrial	20.25	Semi-natural	2007	12	1	137	113	number of seeds per ramet	percentage of grassland habitat in 1994|percentage of grassland habitat in 1938|percentage of grassland habitat in 1835|diversity of the landscape matrix in 1994|diversity of the landscape matrix in 1938	presence/absence	Purschke, O., Sykes, M. T., Reitalu, T., Poschlod, P., & Prentice, H. C. (2012). Linking landscape history and dispersal traits in grassland plant communities. Oecologia, 168(3), 773–783. doi:10.1007/s00442-011-2142-6
Rachello2007	Coral reefs, Jakarta, Indonesia	41	Corals	Marine	2242	Mixed	1995	47	5	93	27	Shape category|Shape category b|Corallites-calice-valleys categoreis|Corallite size|Colony form	Distance to Jakarta|Distance to mainland|Algal assemblage cover|Coraline algae cover|Turf algae cover	number of colonies	Rachello-Dolmen, P. G., & Cleary, D. F. R. (2007). Relating coral species traits to environmental conditions in the Jakarta Bay/Pulau Seribu reef system, Indonesia. Estuarine, Coastal and Shelf Science, 73(3), 816–826. doi:10.1016/j.ecss.2007.03.017
Raevel2012	Montpellier district, Mediterranean vertical outcrops	42	Plants	Terrestrial	1886	Semi-natural	2008–2009	3	7	97	52	vegetative height|specific leaf area|seed mass|start of flowering|seed dispersal mode	AgeClass|Height|Slope	number of individuals	Raevel, V., Violle, C., & Munoz, F. (2012). Mechanisms of ecological succession: insights from plant functional strategies. Oikos, 121(11), 1761–1770. doi:10.1111/j.1600-0706.2012.20261.x
Ribera2001	Scotland	43	Beetles	Terrestrial	78772	Mixed	1995–1997	19	20	68	87	diameter of the eye|length of the antenna|maximum width of the pronotum|maximum depth of the pronotum|maximum width of the elytra	Land use type|Texture|organic content|soil pH|available P	number of individuals	Ribera, I., Dolédec, S., Downie, I. S., & Foster, G. N. (2001). Effect of Land Disturbance and Stress on Species Traits of Ground Beetle Assemblages. Ecology, 82(4), 1112–1129. doi:10.1890/0012-9658(2001)082[1112:EOLDAS]2.0.CO;2
Robinson2014	Various habitats, protected reserves, Prague region, Czech Republic	44	Butterflies	Terrestrial	260	Semi-natural	2003–2004	7	6	71	20	Average wing length|Eggs per batch|Voltinism|Diapause strategy|Diet breadth|Fligth period	Habitat area|Shape complexity|Edge permeability|Area of open habitat|Proportion of open habitat	number of individuals	Robinson, N., Kadlec, T., Bowers, M. D., & Guralnick, R. P. (2014). Integrating species traits and habitat characteristics into models of butterfly diversity in a fragmented ecosystem. Ecological Modelling, 281, 15–25. doi:10.1016/j.ecolmodel.2014.01.022 Kadlec, T., Benes, J., Jarosik, V., & Konvicka, M. (2008). Revisiting urban refuges: Changes of butterfly and burnet fauna in Prague reserves over three decades. Landscape and Urban Planning, 85(1), 1–11. doi:10.1016/j.landurbplan.2007.07.007 Konvicka, M., & Kadlec, T. (2011). How to increase the value of urban areas for butterfly conservation? A lesson from Prague nature reserves and parks. European Journal of Entomology, 108(2), 219–229. doi:10.14411/eje.2011.030
Robroek2017a	Peat bogs, Western Europe	45	Vascular plants	Terrestrial	3800000	Natural	2010–2011	9	5	15	56	SLA|Canopy_height|LDMC|Seed_mass|Seed_number	Altitude|Bioclimatic|Mean annual temperature|Seasonality in temperature|Mean annual precipitation	number of individuals	Robroek, B. J. M., Jassey, V. E. J., Payne, R. J., Martí, M., Bragazza, L., Bleeker, A., … Verhoeven, J. T. A. (2017). Taxonomic and functional turnover are decoupled in European peat bogs. Nature Communications, 8(1161). doi:10.1038/s41467-017-01350-5
Robroek2017b	Peat bogs, Western Europe	45	Bryophytes	Terrestrial	3800000	Natural	2010–2011	9	12	10	56	SLA|Canopy_height|LDMC|Seed_mass|Seed_number	Altitude|Bioclimatic|Mean annual temperature|Seasonality in temperature|Mean annual precipitation	number of individuals	Robroek, B. J. M., Jassey, V. E. J., Payne, R. J., Martí, M., Bragazza, L., Bleeker, A., … Verhoeven, J. T. A. (2017). Taxonomic and functional turnover are decoupled in European peat bogs. Nature Communications, 8(1161). doi:10.1038/s41467-017-01350-5
Shieh2012	Wu Stream, central Taiwan	46	Macroinvertebrates	Freshwater	696	Mixed	2005–2006	11	38	30	48	Collector-gatherer|Shredder|maximum body size|body flexibility|Temporary attachment	Water temperature|Conductivity|Alkalinity|Sulfate|Elevation	number of individuals	Shieh, S.-H., Wang, L.-K., & Hsiao, W.-F. (2012). Shifts in functional traits of aquatic insects along a subtropical stream in Taiwan. Zoological Studies, 51(7), 1051–1065.
Spake2016	Coniferous plantations, UK	47	Beetles	Terrestrial	95000	Forestry	1995–1997	9	6	51	44	Body length|Adult feeding guild|Hind-wing morphology|Activity pattern|Adult habitat affinity	Chronosequence stage|Crop type|Bioclimatic zone|Percentage cover of open semi-natural area|Field, 10 cm – 1.9 m high	number of individuals	Spake, R., Barsoum, N., Newton, A. C., & Doncaster, C. P. (2016). Drivers of the composition and diversity of carabid functional traits in UK coniferous plantations. Forest Ecology and Management, 359, 300–308. doi:10.1016/j.foreco.2015.10.008
Stanko2014	Slovakia	48	Flea	Terrestrial	12000	Agricultural	1986, 1990	16	6	27	13	Abundance|Host number|Continental phylogenetic distinctness|Regional phylogenetic distinctness|Place of living	Mean altitude|Minimal altitude|Maximal altitude|NDVI for autumn|NDVI for spring	number of individuals	Krasnov, B. R., Shenbrot, G. I., Khokhlova, I. S., Stanko, M., Morand, S., & Mouillot, D. (2015). Assembly rules of ectoparasite communities across scales: combining patterns of abiotic factors, host composition, geographic space, phylogeny and traits. Ecography, 38(2), 184–197. doi:10.1111/ecog.00915
Urban2004a	Ponds, 200-ha section of the Yale-Myers Research Station in Union, Connecticut, USA	49	Macroinvertebrates	Freshwater	2	Mixed	1999–2000	6	14	71	14	dispersal mode|trophic category	Pond permanence|Loge Area|Max. Depth|Percent Vegetation structure|Percent canopy cover	presence/absence	Urban, M. C. (2004). Disturbance heterogeneity determines freshwater metacommunity structure. Ecology, 85(11), 2971–2978. Retrieved from http://www.esajournals.org/doi/abs/10.1890/03-0631
Urban2004b	Ponds, 200-ha section of the Yale-Myers Research Station in Union, Connecticut, USA	49	Amphibians	Freshwater	2	Mixed	1999–2000	6	2	7	11	dispersal mode|trophic category	Pond permanence|Loge Area|Max. Depth|Percent Vegetation structure|Percent canopy cover	presence/absence	Urban, M. C. (2004). Disturbance heterogeneity determines freshwater metacommunity structure. Ecology, 85(11), 2971–2978. Retrieved from http://www.esajournals.org/doi/abs/10.1890/03-0631
vanKlink2017	Low intensity hay meadows, Swiss Plateau, Switzerland	50	Plants	Terrestrial	12154	Agricultural	2014–2015	11	5	129	35	Canopy mean size|Canopy maximal size|Flowering start|Flowering end|Flowering mean	Landscape unit|Treatment|Total annual precipitation|Elevation|Forest	percentage cover	van Klink, R., Boch, S., Buri, P., Rieder, N. S., Humbert, J.-Y., & Arlettaz, R. (2017). No detrimental effects of delayed mowing or uncut grass refuges on plant and bryophyte community structure and phytomass production in low-intensity hay meadows. Basic and Applied Ecology, 20, 1–9. doi:10.1016/j.baae.2017.02.003
vanKlink2018a	Low intensity hay meadows, Swiss Plateau, Switzerland	50	Bees	Terrestrial	12154	Agricultural	2014–2015	11	7	46	35	Nesting guild|Larval substrate	Landscape unit|Treatment|Total annual precipitation|Elevation|Forest	Number of individuals	van Klink, R., Menz, M. H. M., Baur, H., Dosch, O., Kühne, I., Lischer, L., … Humbert, J.-Y. (2019). Larval and phenological traits predict invertebrate community response to mowing regime manipulations. Ecological Applications.
vanKlink2018b	Low intensity hay meadows, Swiss Plateau, Switzerland	50	Moths	Terrestrial	12154	Agricultural	2014–2015	11	7	87	35	Family|Minimal winspan|Maximal wingspan|Larval substrate	Landscape unit|Treatment|Total annual precipitation|Elevation|Forest	Number of individuals	van Klink, R., Menz, M. H. M., Baur, H., Dosch, O., Kühne, I., Lischer, L., … Humbert, J.-Y. (2019). Larval and phenological traits predict invertebrate community response to mowing regime manipulations. Ecological Applications.
vanKlink2018c	Low intensity hay meadows, Swiss Plateau, Switzerland	50	Ground beetles	Terrestrial	12154	Agricultural	2014–2015	11	7	60	33	Minimal size|Maximal size|Hind wing development|Trophic level|Hibernation	Landscape unit|Treatment|Total annual precipitation|Elevation|Forest	Number of individuals	van Klink, R., Menz, M. H. M., Baur, H., Dosch, O., Kühne, I., Lischer, L., … Humbert, J.-Y. (2019). Larval and phenological traits predict invertebrate community response to mowing regime manipulations. Ecological Applications.
vanKlink2018d	Low intensity hay meadows, Swiss Plateau, Switzerland	50	Rove beetles	Terrestrial	12154	Agricultural	2014–2015	11	4	82	32	Minimal body length|Maximal body length|Humidity preference	Landscape unit|Treatment|Total annual precipitation|Elevation|Forest	Number of individuals	van Klink, R., Menz, M. H. M., Baur, H., Dosch, O., Kühne, I., Lischer, L., … Humbert, J.-Y. (2019). Larval and phenological traits predict invertebrate community response to mowing regime manipulations. Ecological Applications.
vanKlink2018e	Low intensity hay meadows, Swiss Plateau, Switzerland	50	Hoverflies	Terrestrial	12154	Agricultural	2014–2015	11	6	26	35	Minimum body size|Maximum body size|Start of adult activitiy|End of activity|Larval substrate	Landscape unit|Treatment|Total annual precipitation|Elevation|Forest	Number of individuals	van Klink, R., Menz, M. H. M., Baur, H., Dosch, O., Kühne, I., Lischer, L., … Humbert, J.-Y. (2019). Larval and phenological traits predict invertebrate community response to mowing regime manipulations. Ecological Applications.
Villeger2012a	Estuarine ecosystem,Terminos Lagoon, Gulf of Mexico, Mexico	51	Fish	Marine	3360	Semi-natural	May-03	4	16	45	35	Mass|Oral gape surface|Oral gape shape|Oral gape position|Gill raker length	Depth|Transparency|Salinity|Dissolved oxygen	biomass	Villéger, S., Miranda, J. R., Hernandez, D. F., & Mouillot, D. (2012). Low Functional β-Diversity Despite High Taxonomic β-Diversity among Tropical Estuarine Fish Communities. PLOS ONE, 7(7), e40679. doi:10.1371/journal.pone.0040679
Villeger2012b	Estuarine ecosystem,Terminos Lagoon, Gulf of Mexico, Mexico	51	Fish	Marine	3360	Semi-natural	Jul-03	4	16	48	34	Mass|Oral gape surface|Oral gape shape|Oral gape position|Gill raker length	Depth|Transparency|Salinity|Dissolved oxygen	biomass	Villéger, S., Miranda, J. R., Hernandez, D. F., & Mouillot, D. (2012). Low Functional β-Diversity Despite High Taxonomic β-Diversity among Tropical Estuarine Fish Communities. PLOS ONE, 7(7), e40679. doi:10.1371/journal.pone.0040679
Villeger2012c	Estuarine ecosystem,Terminos Lagoon, Gulf of Mexico, Mexico	51	Fish	Marine	3360	Semi-natural	Nov-03	4	16	47	34	Mass|Oral gape surface|Oral gape shape|Oral gape position|Gill raker length	Depth|Transparency|Salinity|Dissolved oxygen	biomass	Villéger, S., Miranda, J. R., Hernandez, D. F., & Mouillot, D. (2012). Low Functional β-Diversity Despite High Taxonomic β-Diversity among Tropical Estuarine Fish Communities. PLOS ONE, 7(7), e40679. doi:10.1371/journal.pone.0040679
Villeger2012d	Estuarine ecosystem,Terminos Lagoon, Gulf of Mexico, Mexico	51	Fish	Marine	3360	Semi-natural	May-06	4	16	43	35	Mass|Oral gape surface|Oral gape shape|Oral gape position|Gill raker length	Depth|Transparency|Salinity|Dissolved oxygen	biomass	Villéger, S., Miranda, J. R., Hernandez, D. F., & Mouillot, D. (2012). Low Functional β-Diversity Despite High Taxonomic β-Diversity among Tropical Estuarine Fish Communities. PLOS ONE, 7(7), e40679. doi:10.1371/journal.pone.0040679
Villeger2012e	Estuarine ecosystem,Terminos Lagoon, Gulf of Mexico, Mexico	51	Fish	Marine	3360	Semi-natural	Jul-06	4	16	46	35	Mass|Oral gape surface|Oral gape shape|Oral gape position|Gill raker length	Depth|Transparency|Salinity|Dissolved oxygen	biomass	Villéger, S., Miranda, J. R., Hernandez, D. F., & Mouillot, D. (2012). Low Functional β-Diversity Despite High Taxonomic β-Diversity among Tropical Estuarine Fish Communities. PLOS ONE, 7(7), e40679. doi:10.1371/journal.pone.0040679
Westgate2012	Eucalypt forest, Booderee National Park, Australia	52	Amphibians	Terrestrial	98	Natural	2007–2008	6	2	12	43	Ability to climb|Ability to burrow	Hydroperiod|Logarithm of pond width|Logarithm of forest percentage|Logarithm of number of trees|Mean interval of fire return	presence/absence	Westgate, M. J., Driscoll, D. A., & Lindenmayer, D. B. (2012). Can the intermediate disturbance hypothesis and information on species traits predict anuran responses to fire? Oikos, 121(10), 1516–1524. doi:10.1111/j.1600-0706.2011.19863.x
Yates2014	Pasture vs remnant vegetation, North east of New South Wales, Australia	53	Ants	Terrestrial	45500	Mixed	2007	9	11	123	18	Minimum inter-eye distance|eye width|head length|mandible length|top tooth length	habitat type|soil carbon-nitrogen ratio|soil p |herb cover|leaf litter cover|bare ground cover|Average ambient daily temperaturelong|lat	number of individuals	Yates, M. L., Andrew, N. R., Binns, M., & Gibb, H. (2014). Morphological traits: predictable responses to macrohabitats across a 300 km scale. PeerJ, 2, e271. doi:10.7717/peerj.271

**Online-only Table 2:** Original dataset citations.

DatasetName	Database	References (original studies/original repository)
Bagaria2012	CESTES	Bagaria, G., Pino, J., Rodà, F. & Guardiola, M. Species traits weakly involved in plant responses to landscape properties in Mediterranean grasslands. *Journal of Vegetation Science* **23**, 432–442 (2012).
Barbaro2009a	CESTES	Barbaro, L. & van Halder, I. Linking bird, carabid beetle and butterfly life‐history traits to habitat fragmentation in mosaic landscapes. *Ecography* **32**, 321–333 (2009).
Barbaro2009b	CESTES	Barbaro, L. & van Halder, I. Linking bird, carabid beetle and butterfly life‐history traits to habitat fragmentation in mosaic landscapes. *Ecography* **32**, 321–333 (2009).
Barbaro2012	CESTES	Barbaro, L., Brockerhoff, E. G., Giffard, B. & van Halder, I. Edge and area effects on avian assemblages and insectivory in fragmented native forests. *Landscape Ecology* **27**, 1451–1463 (2012).
Barbaro2017	CESTES	Barbaro, L. *et al*. Avian pest control in vineyards is driven by interactions between bird functional diversity and landscape heterogeneity. *J Appl Ecol* **54**, 500–508 (2017).
Bartonova2016	CESTES	Bartonova, A., Benes, J., Fric, Z. F., Chobot, K. & Konvicka, M. How universal are reserve design rules? A test using butterflies and their life history traits. *Ecography* **39**, 456–464 (2016).
Bonada2007S	CESTES	Bonada, N., Rieradevall, M. & Prat, N. Macroinvertebrate community structure and biological traits related to flow permanence in a Mediterranean river network. *Hydrobiologia* **589**, 91–106 (2007).
Bonada2007W	CESTES	Bonada, N., Rieradevall, M. & Prat, N. Macroinvertebrate community structure and biological traits related to flow permanence in a Mediterranean river network. *Hydrobiologia* **589**, 91–106 (2007).
BrindAmour2011a	CESTES	Brind’Amour, A., Boisclair, D., Dray, S. & Legendre, P. Relationships between species feeding traits and environmental conditions in fish communities: a three-matrix approach. *Ecological Applications* **21**, 363–377 (2011).
BrindAmour2011b	CESTES	Brind’Amour, A., Boisclair, D., Dray, S. & Legendre, P. Relationships between species feeding traits and environmental conditions in fish communities: a three-matrix approach. *Ecological Applications* **21**, 363–377 (2011).
Campos2018	CESTES	Campos, R. de, Lansac-Tôha, F. M., Conceição, E. de O. da, Martens, K. & Higuti, J. Factors affecting the metacommunity structure of periphytic ostracods (Crustacea, Ostracoda): a deconstruction approach based on biological traits. *Aquat Sci* **80**, 16 (2018).
Carvalho2015	CESTES	Carvalho, R. A. & Tejerina-Garro, F. L. The influence of environmental variables on the functional structure of headwater stream fish assemblages: a study of two tropical basins in Central Brazil. *Neotropical Ichthyology* **13**, 349–360 (2015).
Castro2010	CESTES	Castro, H., Lehsten, V., Lavorel, S. & Freitas, H. Functional response traits in relation to land use change in the Montado. *Agriculture, Ecosystems & Environment* **137**, 183–191 (2010).
Charbonnier2016a	CESTES	Charbonnier, Y. M. *et al*. Bat and bird diversity along independent gradients of latitude and tree composition in European forests. *Oecologia* **182**, 529–537 (2016).
Charbonnier2016b	CESTES	Charbonnier, Y. M. *et al*. Bat and bird diversity along independent gradients of latitude and tree composition in European forests. *Oecologia* **182**, 529–537 (2016).
Chmura2016	CESTES	Chmura, D., Żarnowiec, J. & Staniaszek-Kik, M. Interactions between plant traits and environmental factors within and among montane forest belts: A study of vascular species colonising decaying logs. *Forest Ecology and Management* **379**, 216–225 (2016).
Choler2005	CESTES	Dray, S. & Dufour, A.-B. The ade4 package: implementing the duality diagram for ecologists. *Journal of Statistical Software* 1–20 (2007). Choler, P. Consistent Shifts in Alpine Plant Traits along a Mesotopographical Gradient. *Arctic, Antarctic, and Alpine Research* **37**, 444–453 (2005).
ChongSeng2012a	CESTES	Chong-Seng, K. M., Mannering, T. D., Pratchett, M. S., Bellwood, D. R. & Graham, N. A. J. The Influence of Coral Reef Benthic Condition on Associated Fish Assemblages. *PLOS ONE* **7**, e42167 (2012).
ChongSeng2012b	CESTES	Chong-Seng, K. M., Mannering, T. D., Pratchett, M. S., Bellwood, D. R. & Graham, N. A. J. The Influence of Coral Reef Benthic Condition on Associated Fish Assemblages. *PLOS ONE* **7**, e42167 (2012).
Cleary2007a	CESTES	Cleary, D. F. R. *et al*. Bird species and traits associated with logged and unlogged forest in Borneo. *figshare* 10.6084/m9.figshare.c.3293726.v1 (2016) Cleary, D. F. R. *et al*. Bird species and traits associated with logged and unlogged forest in Borneo. Ecological Applications 17, 1184–1197 (2007).
Cleary2007b	CESTES	Cleary, D. F. R. & Renema, W. Relating species traits of foraminifera to environmental variables in the Spermonde Archipelago, Indonesia. Marine Ecology Progress Series 334, 73–82 (2007).
Cleary2016	CESTES	Cleary, D. F. R. *et al*. Variation in the composition of corals, fishes, sponges, echinoderms, ascidians, molluscs, foraminifera and macroalgae across a pronounced in-to-offshore environmental gradient in the Jakarta Bay–Thousand Islands coral reef complex. *Marine Pollution Bulletin* **110**, 701–717 (2016).
Cornwell2009	CESTES	Cornwell, W. K. & Ackerly, D. D. Community assembly and shifts in plant trait distributions across an environmental gradient in coastal California. *Ecological Monographs* **79**, 109–126 (2009).
Diaz2008	CESTES	Mellado-Diaz, A., Luisa Suarez Alonso, M. & Rosario Vidal-Abarca Gutierrez, M. Biological traits of stream macroinvertebrates from a semi-arid catchment: patterns along complex environmental gradients. *Freshwater Biology* **53**, 1–21 (2008).
Doledec1996	CESTES	Dray, S. & Dufour, A.-B. The ade4 package: implementing the duality diagram for ecologists. Journal of Statistical Software 1–20 (2007). Dolédec, S., Chessel, D., Braak, C. J. F. ter & Champely, S. Matching species traits to environmental variables: a new three-table ordination method. *Environ Ecol Stat* **3**, 143–166 (1996).
Drew2017	CESTES	Drew, J. A. & Amatangelo, K. L. Community assembly of coral reef fishes along the Melanesian biodiversity gradient. figshare 10.1371/journal.pone.0186123 (2017). Drew, J. A. & Amatangelo, K. L. Community assembly of coral reef fishes along the Melanesian biodiversity gradient. *PLOS ONE* **12**, (2017).
Dziock2011	CESTES	Dziock, F. *et al*. Reproducing or dispersing? Using trait based habitat templet models to analyse Orthoptera response to flooding and land use. *Agriculture, Ecosystems & Environment* **145**, 85–94 (2011). Klaiber, J. *et al*. Fauna Indicativa. (Eidg. Forschungsanstalt für Wald, Schnee und Landschaft WSL, CH-Birmensdorf, 2017).
Eallonardo2013	CESTES	Eallonardo, A. S., Leopold, D. J., Fridley, J. D. & Stella, J. C. Salinity tolerance and the decoupling of resource axis plant traits. *Journal of Vegetation Science* **24**, 365–374 (2013).
Farneda2015	CESTES	Farneda, F. Z. *et al*. Trait-related responses to habitat fragmentation in Amazonian bats. *Journal of Applied Ecology* **52**, 1381–1391 (2015).
Frenette2012a	CESTES	Frenette-Dussault, C., Shipley, B., Léger, J.-F., Meziane, D. & Hingrat, Y. Functional structure of an arid steppe plant community reveals similarities with Grime’s C-S-R theory. *Journal of Vegetation Science* **23**, 208–222 (2012).
Frenette2012b	CESTES	Frenette-Dussault, C., Shipley, B., Léger, J.-F., Meziane, D. & Hingrat, Y. Functional structure of an arid steppe plant community reveals similarities with Grime’s C-S-R theory. *Journal of Vegetation Science* **23**, 208–222 (2012).
Frenette2013	CESTES	Frenette-Dussault, C., Shipley, B. & Hingrat, Y. Linking plant and insect traits to understand multitrophic community structure in arid steppes. *Functional Ecology* **27**, 786–792 (2013).
Fried2012	CESTES	Fried, G., Kazakou, E. & Gaba, S. Trajectories of weed communities explained by traits associated with species’ response to management practices. *Agriculture, Ecosystems & Environment* **158**, 147–155 (2012).
Gallardo2009	CESTES	Gallardo, B., Gascon, S., Garcia, M. & Comin, F. A. Testing the response of macroinvertebrate functional structure and biodiversity to flooding and confinement. *Journal of limnology* **68**, 315–326 (2009).
Gibb2015	CESTES	Gibb, H. *et al*. Responses of foliage-living spider assemblage composition and traits to a climatic gradient in Themeda grasslands: Spider Traits and Climatic Gradients. *Austral Ecology* **40**, 225–237 (2015).
Goncalves2010	CESTES	Gonçalves-Souza, T., Brescovit, A. D., de C. Rossa-Feres, D. & Romero, G. Q. Bromeliads as biodiversity amplifiers and habitat segregation of spider communities in a Neotropical rainforest. *The Journal of Arachnology* **38**, 270–279 (2010).
Goncalves2014a	CESTES	Gonçalves-Souza, T., Romero, G. Q. & Cottenie, K. Metacommunity versus Biogeography: A Case Study of Two Groups of Neotropical Vegetation-Dwelling Arthropods. *PLOS ONE* **9**, e115137 (2014).
Goncalves2014b	CESTES	Gonçalves-Souza, T., Romero, G. Q. & Cottenie, K. Metacommunity versus Biogeography: A Case Study of Two Groups of Neotropical Vegetation-Dwelling Arthropods. *PLOS ONE* **9**, e115137 (2014).
Jamil2013	CESTES	Jamil, T., Ozinga, W. A., Kleyer, M. & ter Braak, C. J. F. Selecting traits that explain species-environment relationships: a generalized linear mixed model approach. *Journal of Vegetation Science* **24**, 988–1000 (2013).
Jeliazkov2013	CESTES	Jeliazkov, A. Scale-effects in agriculture-environment-biodiversity relationships. (Université Pierre et Marie Curie, 2013). Retrieved from http://www.sudoc.fr/180446460
Jeliazkov2014	CESTES	Jeliazkov, A. *et al*. Level-dependence of the relationships between amphibian biodiversity and environment in pond systems within an intensive agricultural landscape. *Hydrobiologia* **723**, 7–23 (2014).
Krasnov2015	CESTES	Krasnov, B. R. *et al*. Assembly rules of ectoparasite communities across scales: combining patterns of abiotic factors, host composition, geographic space, phylogeny and traits. *Ecography* **38**, 184–197 (2015).
Lowe2018a	CESTES	Lowe, E. C., Threlfall, C. G., Wilder, S. M. & Hochuli, D. F. Environmental drivers of spider community composition at multiple scales along an urban gradient. *Biodivers Conserv* **27**, 829–852 (2018).
Lowe2018b	CESTES	Lowe, E. C., Threlfall, C. G., Wilder, S. M. & Hochuli, D. F. Environmental drivers of spider community composition at multiple scales along an urban gradient. *Biodivers Conserv* **27**, 829–852 (2018).
Marteinsdottir2014	CESTES	Marteinsdóttir, B. & Eriksson, O. Plant community assembly in semi-natural grasslands and ex-arable fields: a trait-based approach. *Journal of Vegetation Science* **25**, 77–87 (2014).
Meffert2013	CESTES	Meffert, P. J. & Dziock, F. The influence of urbanisation on diversity and trait composition of birds. *Landscape Ecology* **28**, 943–957 (2013).
Ossola2015	CESTES	Ossola, A., Nash, M. A., Christie, F. J., Hahs, A. K. & Livesley, S. J. Urban habitat complexity affects species richness but not environmental filtering of morphologically-diverse ants. *PeerJ* **3**, e1356 (2015).
Pakeman2011	CESTES	Pakeman, R. J. Multivariate identification of plant functional response and effect traits in an agricultural landscape. *Ecology* **92**, 1353–1365 (2011).
Pavoine2011	CESTES	Dray, S. Dufour, A.-B. The ade4 package: implementing the duality diagram for ecologists. *Journal of Statistical Software* 1–20 (2007). de Bélair, Gérard and Bencheikh-Lehocine, Mahmoud (1987) Composition et déterminisme de la végétation d'une plaine côtière marécageuse: La Mafragh (Annaba, Algérie). *Bulletin d'Ecologie*, **18**(4), 393–407. Pavoine, S., Vela, E., Gachet, S., de Bélair, G. & Bonsall, M. B. Linking patterns in phylogeny, traits, abiotic variables and space: a novel approach to linking environmental filtering and plant community assembly: Multiple data in community organization. *Journal of Ecology* **99**, 165–175 (2011).
Pekin2011	CESTES	Pekin, B. K., Wittkuhn, R. S., Boer, M. M., Macfarlane, C. & Grierson, P. F. Plant functional traits along environmental gradients in seasonally dry and fire-prone ecosystem. *Journal of Vegetation Science* **22**, 1009–1020 (2011).
Pomati2013	CESTES	Pomati, F. *et al*. Individual Cell Based Traits Obtained by Scanning Flow-Cytometry Show Selection by Biotic and Abiotic Environmental Factors during a Phytoplankton Spring Bloom. *PLOS ONE* **8**, e71677 (2013).
Purschke2012a	CESTES	Purschke, O., Sykes, M. T., Reitalu, T., Poschlod, P. & Prentice, H. C. Linking landscape history and dispersal traits in grassland plant communities. *Oecologia* **168**, 773–783 (2012).
Purschke2012b	CESTES	Purschke, O., Sykes, M. T., Reitalu, T., Poschlod, P. & Prentice, H. C. Linking landscape history and dispersal traits in grassland plant communities. *Oecologia* **168**, 773–783 (2012).
Purschke2012c	CESTES	Purschke, O., Sykes, M. T., Reitalu, T., Poschlod, P. & Prentice, H. C. Linking landscape history and dispersal traits in grassland plant communities. *Oecologia* **168**, 773–783 (2012).
Purschke2012d	CESTES	Purschke, O., Sykes, M. T., Reitalu, T., Poschlod, P. & Prentice, H. C. Linking landscape history and dispersal traits in grassland plant communities. *Oecologia* **168**, 773–783 (2012).
Purschke2012e	CESTES	Purschke, O., Sykes, M. T., Reitalu, T., Poschlod, P. & Prentice, H. C. Linking landscape history and dispersal traits in grassland plant communities. *Oecologia* **168**, 773–783 (2012).
Rachello2007	CESTES	Rachello-Dolmen, P. G. & Cleary, D. F. R. Relating coral species traits to environmental conditions in the Jakarta Bay/Pulau Seribu reef system, Indonesia. *Estuarine, Coastal and Shelf Science* **73**, 816–826 (2007).
Raevel2012	CESTES	Raevel, V., Violle, C. & Munoz, F. Mechanisms of ecological succession: insights from plant functional strategies. *Oikos* **121**, 1761–1770 (2012).
Ribera2001	CESTES	Ribera, I., Dolédec, S., Downie, I. S. & Foster, G. N. Effect of Land Disturbance and Stress on Species Traits of Ground Beetle Assemblages. *Ecology* **82**, 1112–1129 (2001).
Robinson2014	CESTES	Robinson, N., Kadlec, T., Bowers, M. D. & Guralnick, R. P. Integrating species traits and habitat characteristics into models of butterfly diversity in a fragmented ecosystem. *Ecological Modelling* **281**, 15–25 (2014). Kadlec, T., Benes, J., Jarosik, V. & Konvicka, M. Revisiting urban refuges: Changes of butterfly and burnet fauna in Prague reserves over three decades. *Landscape and Urban Planning* **85**, 1–11 (2008). Konvicka, M. & Kadlec, T. How to increase the value of urban areas for butterfly conservation? A lesson from Prague nature reserves and parks. *European Journal of Entomology* **108**, 219–229 (2011).
Robroek2017a	CESTES	Robroek, B. *et al*. Data from: Taxonomic and functional turnover are decoupled in European peat bogs. *Dryad Digitial Repository* https://doi.org/10.5061/dryad.g1pk3 (2017) Robroek, B. J. M. *et al*. Taxonomic and functional turnover are decoupled in European peat bogs. *Nature Communications* **8**, (2017).
Robroek2017b	CESTES	Robroek, B. *et al*. Data from: Taxonomic and functional turnover are decoupled in European peat bogs. *Dryad Digitial Repository* https://doi.org/10.5061/dryad.g1pk3 (2017) Robroek, B. J. M. *et al*. Taxonomic and functional turnover are decoupled in European peat bogs. *Nature Communications* **8**, (2017).
Shieh2012	CESTES	Shieh, S.-H., Wang, L.-K. & Hsiao, W.-F. Shifts in Functional Traits of Aquatic Insects along a Subtropical Stream in Taiwan. *Zoological Studies* **51**, 1051–1065 (2012).
Spake2016	CESTES	Spake, R., Barsoum, N., Newton, A. C. & Doncaster, C. P. Drivers of the composition and diversity of carabid functional traits in UK coniferous plantations. *Forest Ecology and Management* **359**, 300–308 (2016).
Stanko2014	CESTES	Krasnov, B. R. *et al*. Assembly rules of ectoparasite communities across scales: combining patterns of abiotic factors, host composition, geographic space, phylogeny and traits. *Ecography* **38**, 184–197 (2015).
Urban2004a	CESTES	Urban, M. C. Disturbance heterogeneity determines freshwater metacommunity structure. *Ecology* **85**, 2971–2978 (2004).
Urban2004b	CESTES	Urban, M. C. Disturbance heterogeneity determines freshwater metacommunity structure. *Ecology* **85**, 2971–2978 (2004).
vanKlink2017	CESTES	van Klink, R. *et al*. No detrimental effects of delayed mowing or uncut grass refuges on plant and bryophyte community structure and phytomass production in low-intensity hay meadows. *Basic and Applied Ecology* **20**, 1–9 (2017).
vanKlink2018a	CESTES	van Klink, R. *et al*. Larval and phenological traits predict invertebrate community response to mowing regime manipulations. *Ecological Applicationse* **e01900** (2019).
vanKlink2018b	CESTES	van Klink, R. *et al*. Larval and phenological traits predict invertebrate community response to mowing regime manipulations. *Ecological Applicationse* **e01900** (2019).
vanKlink2018c	CESTES	van Klink, R. *et al*. Larval and phenological traits predict invertebrate community response to mowing regime manipulations. *Ecological Applicationse* **e01900** (2019).
vanKlink2018d	CESTES	van Klink, R. *et al*. Larval and phenological traits predict invertebrate community response to mowing regime manipulations. *Ecological Applicationse* **e01900** (2019).
vanKlink2018e	CESTES	van Klink, R. *et al*. Larval and phenological traits predict invertebrate community response to mowing regime manipulations. *Ecological Applicationse* **e01900** (2019).
Villeger2012a	CESTES	Villéger, S., Miranda, J. R., Hernandez, D. F. & Mouillot, D. Low Functional β-Diversity Despite High Taxonomic β-Diversity among Tropical Estuarine Fish Communities. *PLOS ONE* **7**, e40679 (2012).
Villeger2012b	CESTES	Villéger, S., Miranda, J. R., Hernandez, D. F. & Mouillot, D. Low Functional β-Diversity Despite High Taxonomic β-Diversity among Tropical Estuarine Fish Communities. *PLOS ONE* **7**, e40679 (2012).
Villeger2012c	CESTES	Villéger, S., Miranda, J. R., Hernandez, D. F. & Mouillot, D. Low Functional β-Diversity Despite High Taxonomic β-Diversity among Tropical Estuarine Fish Communities. *PLOS ONE* **7**, e40679 (2012).
Villeger2012d	CESTES	Villéger, S., Miranda, J. R., Hernandez, D. F. & Mouillot, D. Low Functional β-Diversity Despite High Taxonomic β-Diversity among Tropical Estuarine Fish Communities. *PLOS ONE* **7**, e40679 (2012).
Villeger2012e	CESTES	Villéger, S., Miranda, J. R., Hernandez, D. F. & Mouillot, D. Low Functional β-Diversity Despite High Taxonomic β-Diversity among Tropical Estuarine Fish Communities. *PLOS ONE* **7**, e40679 (2012).
Westgate2012	CESTES	Westgate, M. J., Driscoll, D. A. & Lindenmayer, D. B. Can the intermediate disturbance hypothesis and information on species traits predict anuran responses to fire? *Oikos* **121**, 1516–1524 (2012).
Yates2014	CESTES	Yates, M. L., Andrew, N. R., Binns, M. & Gibb, H. Morphological traits: predictable responses to macrohabitats across a 300 km scale. *PeerJ* **2**, e271 (2014).
Belskaya2017	ceste	Belskaya, E. A. & Zolotarev, M. P. Changes in the size structure of carabid communities in forest ecosystems under technogenic transformation. *Russian Journal of Ecology* **48**, 152–160 (2017).
Chambers201X	ceste	Unpublished
Cleary2007d	ceste	Cleary, D. F. R. *et al*. Variation in the diversity and composition of benthic taxa as a function of distance offshore, depth and exposure in the Spermonde Archipelago, Indonesia. *Estuarine, Coastal and Shelf Science* **65**, 557–570 (2005). de Voogd, N. J. & Cleary, D. F. R. Relating species traits to environmental variables in Indonesian coral reef sponge assemblages. Mar. Freshwater Res. 58, 240–249 (2007).
Cormont2011	ceste	Cormont, A., Vos, C., van Turnhout, C., Foppen, R. & ter Braak, C. Using life-history traits to explain bird population responses to changing weather variability. *Climate Research* **49**, 59–71 (2011).
Huebner2012	ceste	Huebner, K., Lindo, Z. & Lechowicz, M. J. Post-fire succession of collembolan communities in a northern hardwood forest. *European Journal of Soil Biology* **48**, 59–65 (2012).
Jamil2012a	ceste	Jamil, T., Opdekamp, W., van Diggelen, R. & ter Braak, C. J. F. Trait-Environment Relationships and Tiered Forward Model Selection in Linear Mixed Models. *International Journal of Ecology* **2012**, 1–12 (2012).
Jamil2012b	ceste	Jamil, T., Opdekamp, W., van Diggelen, R. & ter Braak, C. J. F. Trait-Environment Relationships and Tiered Forward Model Selection in Linear Mixed Models. *International Journal of Ecology* **2012**, 1–12 (2012).
Jamil2012c	ceste	Jamil, T., Opdekamp, W., van Diggelen, R. & ter Braak, C. J. F. Trait-Environment Relationships and Tiered Forward Model Selection in Linear Mixed Models. *International Journal of Ecology* **2012**, 1–12 (2012).
Jamil2014	ceste	Jamil, T., Kruk, C. & ter Braak, C. J. F. A unimodal species response model relating traits to environment with application to phytoplankton communities. *PLOS ONE* **9**, e97583 (2014).
Palozzi2017	ceste	Palozzi, J. E. & Lindo, Z. Boreal peat properties link to plant functional traits of ecosystem engineers. *Plant Soil* **418**, 277–291 (2017).

**Online-only Table 3:** Overview of ceste, the non-spatial ancillary to CESTES.

DatasetName	Taxonomic group	Ecosystem and location	Ecosystem type	Level of human disturbance	Extent (km2)	Sampling date(s)/period	nbEnv	nbTra	nbSpe	nbSit	Example of traits	Example of environmental variables	References
Belskaya2017	Insects	Spruce-fir forest, Middle Ural Copper Smelter, Revda, Sverdlovsk oblast, Russia	Terrestrial	Mixed	429	2009, 2013	1	1	54	3 or 10	Bodz size	Pollution level	Belskaya, E. A., & Zolotarev, M. P. (2017). Changes in the size structure of carabid communities in forest ecosystems under technogenic transformation. Russian Journal of Ecology, 48(2), 152–160. doi:10.1134/S1067413617010040
Chambers201X	Plants	Grassland restoration	Terrestrial	Agricultural	NA	NA	9	7	21	24	specific leaf area|height|Nfixer|entomophily|dispersal	moisture|litter|pH|phosphorous|available nitrogen	Unpublished
Cleary2007d	Sponges	Coral reefs, Spermonde Archipelago, Makassar, southwest Sulawesi, Indonesia	Marine	Mixed	2418	1989	16	3	150	30	bioactivity|reproductive mode|growth form	turbidity|mean velocity|mean salinity|mean temperature|exposure to oceanic currents	Cleary, D. F. R., Becking, L. E., de Voogd, N. J., Renema, W., de Beer, M., van Soest, R. W. M., & Hoeksema, B. W. (2005). Variation in the diversity and composition of benthic taxa as a function of distance offshore, depth and exposure in the Spermonde Archipelago, Indonesia. Estuarine, Coastal and Shelf Science, 65(3), 557–570. doi:10.1016/j.ecss.2005.06.025 de Voogd, N. J., & Cleary, D. F. R. (2007). Relating species traits to environmental variables in Indonesian coral reef sponge assemblages. Marine and Freshwater Research, 58(3), 240–249. doi:10.1071/MF06125
Cormont2011	Birds	Marshland, forests, Netherlands	Terrestrial	Natural	41000	1984–2004	20	13	43	21	nest location|mean diet type|development|migration strategy	winter severity|mean temperature of the coldest month|precipitation over non-breeding season|precipitation over breeding season|number of heatwave days	Cormont, A., Vos, C., van Turnhout, C., Foppen, R., & ter Braak, C. (2011). Using life-history traits to explain bird population responses to changing weather variability. Climate Research, 49(1), 59–71. doi:10.3354/cr01007
Huebner2012	Collembola	Fire forest, Gault Nature Reserve Québec, Canada	Terrestrial	Natural	NA	2009	11	35	17	40	shape of dentes|number of eyes|total body length|furcula|pigmentation	ground fire intensity|moisture content	Huebner, K., Lindo, Z., & Lechowicz, M. J. (2012). Post-fire succession of collembolan communities in a northern hardwood forest. European Journal of Soil Biology, 48, 59–65. doi:10.1016/j.ejsobi.2011.10.004
Jamil2012a	Plants	Outdoor mesocosms, floodplain, whole Netherlands (?)	Terrestrial	Experimental	NA	2006	3	45	28	80	seed weight|germination percentage|height of seedlings|mean leaf weight|mean leaf area	treatment of canopy presence|treatment of waterlogging|treatment of mowing	Jamil, T., Kruk, C., & ter Braak, C. J. F. (2014). A unimodal species response model relating traits to environment with application to phytoplankton communities. PLoS ONE, 9(5), e97583. doi:10.1371/journal.pone.0097583
Jamil2012b	Plants	Outdoor mesocosms, floodplain, whole Netherlands (?)	Terrestrial	Experimental	NA	2007	3	45	28	80	seed weight|germination percentage|height of seedlings|mean leaf weight|mean leaf area	treatment of canopy presence|treatment of waterlogging|treatment of mowing	Jamil, T., Kruk, C., & ter Braak, C. J. F. (2014). A unimodal species response model relating traits to environment with application to phytoplankton communities. PLoS ONE, 9(5), e97583. doi:10.1371/journal.pone.0097583
Jamil2012c	Plants	Outdoor mesocosms, floodplain, whole Netherlands (?)	Terrestrial	Experimental	NA	2008	3	45	28	80	seed weight|germination percentage|height of seedlings|mean leaf weight|mean leaf area	treatment of canopy presence|treatment of waterlogging|treatment of mowing	Jamil, T., Kruk, C., & ter Braak, C. J. F. (2014). A unimodal species response model relating traits to environment with application to phytoplankton communities. PLoS ONE, 9(5), e97583. doi:10.1371/journal.pone.0097583
Jamil2014	Phytoplankton	Lakes, South America, Europe, and North America	Freshwater	Mixed	52000000	1999, 2005–2006	13	8	60	201	volume|surface area|maximum linear dimension|aerotopes|flagella	temperature|inorganic suspended solids|water column mix depth|light attenuation coefficient|conductivity	Jamil, T., Opdekamp, W., van Diggelen, R., & ter Braak, C. J. F. (2012). Trait-Environment Relationships and Tiered Forward Model Selection in Linear Mixed Models. International Journal of Ecology, 2012, 1–12. doi:10.1155/2012/947103
Palozzi2017	Plants	White River Experimental Watersheds, Boreal peatland complex, Ontario, Canada	Terrestrial	Experimental	4	2015	15	9	14	10	heigth|leaf area|dry leaf mass|specific leaf area|leaf mass per area	water pH|electrical conductivity|moisture content|phosphate concentration|basal respiration	Palozzi, J. E., & Lindo, Z. (2017). Boreal peat properties link to plant functional traits of ecosystem engineers. Plant and Soil, 418(1–2), 277–291. doi:10.1007/s11104-017-3291-0
