# Author Correction: A global database for metacommunity ecology, integrating species, traits, environment and space

**DOI:** 10.1038/s41597-020-0420-z

**Published:** 2020-03-02

**Authors:** Alienor Jeliazkov, Darko Mijatovic, Stéphane Chantepie, Nigel Andrew, Raphaël Arlettaz, Luc Barbaro, Nadia Barsoum, Alena Bartonova, Elena Belskaya, Núria Bonada, Anik Brind’Amour, Rodrigo Carvalho, Helena Castro, Damian Chmura, Philippe Choler, Karen Chong-Seng, Daniel Cleary, Anouk Cormont, William Cornwell, Ramiro de Campos, Nicole de Voogd, Sylvain Doledec, Joshua Drew, Frank Dziock, Anthony Eallonardo, Melanie J. Edgar, Fábio Farneda, Domingo Flores Hernandez, Cédric Frenette-Dussault, Guillaume Fried, Belinda Gallardo, Heloise Gibb, Thiago Gonçalves-Souza, Janet Higuti, Jean-Yves Humbert, Boris R. Krasnov, Eric Le Saux, Zoe Lindo, Adria Lopez-Baucells, Elizabeth Lowe, Bryndis Marteinsdottir, Koen Martens, Peter Meffert, Andres Mellado-Díaz, Myles H. M. Menz, Christoph F. J. Meyer, Julia Ramos Miranda, David Mouillot, Alessandro Ossola, Robin Pakeman, Sandrine Pavoine, Burak Pekin, Joan Pino, Arnaud Pocheville, Francesco Pomati, Peter Poschlod, Honor C. Prentice, Oliver Purschke, Valerie Raevel, Triin Reitalu, Willem Renema, Ignacio Ribera, Natalie Robinson, Bjorn Robroek, Ricardo Rocha, Sen-Her Shieh, Rebecca Spake, Monika Staniaszek-Kik, Michal Stanko, Francisco Leonardo Tejerina-Garro, Cajo ter Braak, Mark C. Urban, Roel van Klink, Sébastien Villéger, Ruut Wegman, Martin J. Westgate, Jonas Wolff, Jan Żarnowiec, Maxim Zolotarev, Jonathan M. Chase

**Affiliations:** 1 German Centre for Integrative Biodiversity Research (iDiv), Deutscher Platz 5E, 04103 Leipzig, Germany; 2 Department of Computer Science, Martin Luther University Halle-Wittenberg, 06099 Halle, Salle Germany; 3 Department of Evolutionary Biology and Environmental Studies, University of Zürich, Winterthurerstrasse 190, 8057 Zurich, Switzerland; 4 Zoology, University of New England, Armidale, NSW 2351 Australia; 5 Division of Conservation Biology, Institute of Ecology and Evolution, University of Bern, 3012 Bern, Switzerland; 6 Dynafor, INRA-INPT, Univ. Toulouse, Auzeville, France; 7 Centre d’Ecologie et des Sciences de la Conservation (CESCO), Muséum National d’Histoire Naturelle, CNRS, Sorbonne Université, Paris, France; 8 Centre for Ecosystems, Society and Biosecurity, Forest Research, Alice Holt Lodge, Farnham, Surrey GU10 4LH UK; 9 Biology Centre CAS, Czech Academy of Sciences, Institute of Entomology, Branisovska 31, 370 05 Ceske Budejovice, Czech Republic; 10 University of South Bohemia in Ceske Budejovice, Faculty of Science, Branisovska 1760, 370 05 Ceske Budejovice, Czech Republic; 11 Institute of Plant and Animal Ecology, Ural Branch, Russian Academy of Sciences, Eighth March Street 202, Yekaterinburg, 620144 Russia; 12 Grup de Recerca “Freshwater Ecology, Hydrology and Management” (FEHM), Departament de Biologia Evolutiva, Ecologia i Ciències Ambientals, Facultat de Biologia, Institut de Recerca de la Biodiversitat (IRBio), Universitat de Barcelona (UB), Diagonal 643, 08028 Barcelona, Catalonia Spain; 13 Unité Écologie et Modèles pour l’Halieutique, IFREMER, Rue de l’île d’Yeu, B.P. 21105, 44311 Nantes, Cedex 03 France; 14 Departamento de Biologia, Universidade Estadual de Goiás, Campus Palmeiras de Goiás, Palmeiras de Goiás, Goiás, Brazil; 15 Universidade Estadual de Goiás (UEG), Programa de Pós-Graduação em Recursos Naturais do Cerrado (RENAC), Campus de Ciências Exatas e Tecnológicas - Henrique Santillo, BR 153, No. 3105 Fazenda Barreiro do Meio, 75132400 Anápolis, GO Brazil; 16 CFE- Centre for Functional Ecology - Science for People & the Planet, Department of Life Sciences, University of Coimbra, 3000-456 Coimbra, Portugal; 17 Institute of Environmental Protection and Engineering, University of Bielsko-Biala, Willowa 2, 43-309 Bielsko-Biała, Poland; 18 Univ. Grenoble Alpes, Univ. Savoie Mont Blanc, CNRS, LECA, F-38000 Grenoble, France; 19 ARC Centre of Excellence for Coral Reef Studies, James Cook University, Townsville, QLD 4811 Australia; 20 Department of Biology & CESAM, University of Aveiro, Campus de Santiago, 3810-193 Aveiro, Portugal; 21 Tropical Island Sustainable Development Research Center, National Penghu University of Science and Technology, 300 Liu-Ho Rd., Magong City, Penghu 880 Taiwan; 22 Wageningen Environmental Research, Wageningen University & Research, Droevendaalsesteeg 3-3 A, 6708 PB Wageningen, The Netherlands; 23 Ecology and Evolution Research Centre, School of Biological, Earth and Environmental Sciences, UNSW Sydney, Sydney, New South Wales 2052 Australia; 24 Programa de Pós-Graduação em Ecologia de Ambientes Aquáticos Continentais, Núcleo de Pesquisas em Limnologia, Ictiologia, Universidade Estadual de Maringá. Av. Colombo, 5790, CEP, 87020-900 Maringá, PR Brazil; 25 Naturalis Biodiversity Center, Marine Biodiversity, Vondellaan 55, 2332 AA Leiden, The Netherlands; 26 Institute of Environmental Sciences, Environmental Biology Department, Leiden University, Einsteinweg 2, 2333 CC Leiden, The Netherlands; 27 UMR 5023 LEHNA, Université Lyon 1, Université Lyon, Villeurbanne, France; 28 Department of Environmental and Forest Biology. State University of New York, College of Environmental Science and Forestry. 1 Forestry Dr. Syracuse, New York, NY 13210 USA; 29 University of Applied Sciences HTW Dresden, Pillnitzer Platz 2, D-01326 Dresden, Germany; 30 OBG, Part of Ramboll, 400 Andrews St., Suite 710, Rochester, NY 14604 USA; 31 Department of Life Sciences, Natural History Museum, Cromwell Road, London, SW7 5BD UK; 32 Department of Ecology, Federal University of Rio de Janeiro, 21941-902 Rio de Janeiro, Brazil; 33 Biological Dynamics of Forest Fragments Project, National Institute for Amazonian Research and Smithsonian Tropical Research Institute, 69011-970 Manaus, Brazil; 34 Centre for Ecology, Evolution and Environmental Changes, University of Lisbon, 1749-016 Lisbon, Portugal; 35 Instituto EPOMEX, Universidad Autónoma de Campeche, Av. Héroe de Nacozari No. 480, Campus VI de Investigación-UAC, San Francisco de Campeche, 24020 Campeche, México; 36 Institut de recherche en biologie végétale, Montréal, Québec, Canada; 37 Anses, Laboratoire de la Santé des Végétaux, Unité Entomologie et Plantes Invasives, Montferrier-sur-Lez, France; 38 Pyrenean Institute of Ecology (IPE-CSIC). Avda. Montanana, 1005 zaragoza, Spain; 39 Department of Ecology, Environment and Evolution and Centre for Future Landscapes, La Trobe University, Melbourne, Victoria 3086 Australia; 40 Department of Biology, Ecological Synthesis and Biodiversity Conservation Lab, Federal Rural University of Pernambuco, Rio de Janeiro, Brazil; 41 Mitrani Department of Desert Ecology, Swiss Institute for Dryland Environmental and Energy Research, Jacob Blaustein Institutes for Desert Research, Ben-Gurion University of the Negev, Sede-Boqer Campus, Midreshet Ben-Gurion, 8499000 Israel; 42 Department of Biology, The University of Western Ontario, London, Ontario Canada; 43 Granollers Museum of Natural Sciences, 08402 Granollers, Catalonia Spain; 44 Department of Biological Sciences, Macquarie University, Sydney, NSW 2109 Australia; 45 The soil conservation service of Iceland, Gunnarsholt, 851 Hella, Iceland; 46 Royal Belgian Institute of Natural Sciences, Vautierstraat 29, 1000 Brussels, Belgium; 47 University of Ghent, Department of Biology, K.L. Ledeganckstraat 35, 9000 Ghent, Belgium; 48 corvus, Lüchow 2, D-17179 Altkalen, Germany; 49 Departamento de Ecología e Hidrología, Facultad de Biología, Universidad de Murcia, 30100 Murcia, Spain; 50 Present Address: Gerencia de Planificación y Gestión Hídrica. TRAGSATEC. C/Valentín Beato, 6, 28037 Madrid, Spain; 51 Department of Migration and Immuno-ecology, Max Planck Institute for Ornithology, 78315 Radolfzell, Germany; 52 School of Environment and Life Sciences, University of Salford, M5 4WT Salford, UK; 53 MARBEC, Univ Montpellier, CNRS, Ifremer, IRD, Montpellier, France; 54 The James Hutton Institute, Craigiebuckler, Aberdeen, AB15 8QH UK; 55 Istanbul Technical University, Eurasia Institute of Earth Sciences, Istanbul, 34469 Turkey; 56 CREAF, Cerdanyola del Vallés 08193, Spain/UAB, Cerdanyola del Vallés, 08193 Spain; 57 CNRS & Université Paul Sabatier, Laboratoire Évolution et Diversité Biologique, UMR 5174, Bât. 4R1, 118 Route de Narbonne, F-31062 Toulouse, cedex 9 France; 58 Eawag, Swiss Federal Institute of Water Science and Technology, Uberlandstrasse 133, 8600 Dubendorf, Switzerland; 59 Ecology and Conservation Biology, Institute for Plant Sciences, University of Regensburg, D-93040 Regensburg, Germany; 60 Department of Biology, Lund University, Sölvegatan 37, SE 223 62 Lund, Sweden; 61 Department of Physical Geography and Ecosystem Science, Lund University, Sölvegatan 37, SE 223 62 Lund, Sweden; 62 Biodiversity, Department of Biology, Lund University, Sölvegatan 37, SE 223 62 Lund, Sweden; 63 CEFE UMR 5175, CNRS - Université de Montpellier - Université Paul-Valéry Montpellier - EPHE, 1919 route de Mende, F-34293 Montpellier, Cedex 5 France; 64 Institute of Geology, Tallinn University of Technology, Ehitajate tee 5, 19086 Tallinn, Estonia; 65 Institute of Evolutionary Biology (CSIC-Universitat Pompeu Fabra), Passeig Maritim Barceloneta 37, 08003 Barcelona, Spain; 66 National Ecological Observatory Network, 1685 38th Street Suite 100, Boulder, CO 80301 USA; 67 University of Colorado Department of Ecology and Evolutionary Biology, UCB 334, University of Colorado, Boulder, CO 80309 USA; 68 School of Biological Sciences, University of Southampton, Highfield Campus, Southampton, SO17 1BJ UK; 69 Department of Ecological Humanities, Providence University, 200, Sec. 7, Taiwan Boulevard, Shalu Dist., Taichung 43301 Taiwan; 70 School of Geography and Environmental Science, University of Southampton, Highfield Campus, Southampton, SO17 1BJ UK; 71 Department of Geobotany and Plant Ecology, University of Lodz, Banacha 12/16, 90-237 Łódź, Poland; 72 Institute of Parasitology and Institute of Zoology, Slovak Acad. Sci., Loffl erova 10, SK-04001 Kosice, Slovakia; 73 Centro de Biologia Aquática, Escola de Ciências Agrárias e Biológicas, Pontifícia Universidade Católica de Goiás, Campus II. Av. Engler s/n, Jd. Mariliza, Goiânia, Goiás CEP 74885460 Brazil; 74 Laboratório de Biodiversidade, Programa de Pós-Graduação em Sociedade Tecnologia e Meio Ambiente, UniEVANGÉLICA. Avenida Universitária km 3,5, Cidade Universitária, Anápolis, Goiás CEP 75083-515 Brazil; 75 Biometris, Wageningen University & Research, Droevendaalsesteeg 1, 6708 PB Wageningen, The Netherlands; 76 University of Connecticut, 75 N. Eagleville Road, Unit 3043, Storrs, CT 06269 USA; 77 Fenner School of Environment & Society, The Australian National University, Acton, ACT 2601 Australia

Correction to: *Scientific Data* 10.1038/s41597-019-0344-7, published online 08 January 2020

Following publication of this Data Descriptor it was found that the affiliation of Oliver Purschke was stated incorrectly. The correct affiliations are stated below:

Department of Physical Geography and Ecosystem Science, Lund University, Sölvegatan 37, SE-223 62 Lund, Sweden

Biodiversity, Department of Biology, Lund University, Sölvegatan 37, SE-223 62 Lund, Sweden

This has been corrected in both the HTML and PDF versions.

