# Supplementary material for: A global database for metacommunity ecology, integrating species, traits, environment and space
Source: Sci Data. 2020 Jan 8;7:6. doi: 10.1038/s41597-019-0344-7 (PMC6949231; doi:10.1038/s41597-019-0344-7)
Supplement: Supplementary file 1 [file 41597_2019_344_MOESM1_ESM.docx]

How to share your data for the CESTES database?

1. What are the eligibility criteria for integration of your dataset in the CESTES database?
2. How to format your data?
3. How to send your data?
4. How will your contribution be acknowledged?
5. Remarks

# Eligibility criteria

For your dataset to be eligible to integration in the CESTES database, it has to fulfil two criteria.

1. Your dataset has to include the following **4 pieces of DATA information**:

- **comm**: matrix of species (or any taxonomic unit) abundances/counts/density or presences/absences recorded in **multiple sites** (i.e. >= 10 sites)(for other cases, see Remarks)
- **traits**: a corresponding matrix of species trait information, i.e. any trait, be it functional, biological, life-history traits, either quantitative or categorical, functional group, etc., with traits related to the species which are reported in comm
- **envir**: a corresponding matrix of environmental variables in the broad sense of environment, i.e. any type of biotic and abiotic conditions or habitat characteristics relevant to the community of interest according to the original study/project, with variables related to the sites which are reported in comm
- **coord**: a corresponding matrix of spatial coordinates, with the longitude, and the latitude of the sites that are reported in comm.

1. Your dataset has to be **formatted** according to the guidelines provided below.

# Formatting guidelines

Each dataset has to include (see **Figure 1 & 2**):

- the **four data matrices** as described above: comm, traits, envir, and coord
- a **metadata table**: DataKey
- a **species list** splist corresponding to the species taxonomy recorded in comm.


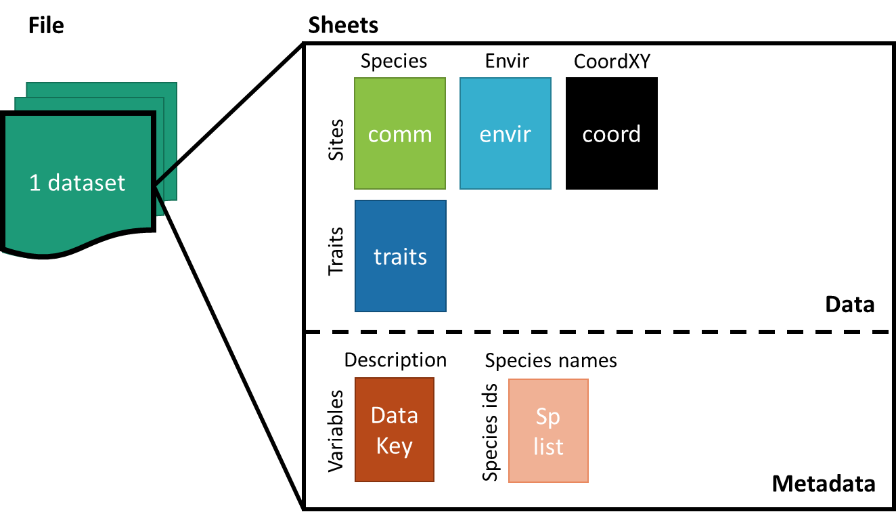


***Figure 1.*** *Structure of the dataset*

1. **Accepted file formats**

Three types of file formats are accepted:

- Comma Separated Value files (.csv, .csv2)
- Spreadsheet files (.ods, .xls, .xlsx)
- R objects (e.g. list, tibble)

1. **Format the four data matrices** (see **Figure 2**)

- **comm**: matrix of species abundances/counts/density or presences/absences, with species in columns and sites in rows
- **traits**: matrix of species trait information, with traits in columns and species in rows
- **envir**: matrix of environmental variables, with variables in columns and sites in rows
- **coord**: matrix of spatial coordinates in the **WGS84 Geographical Coordinate System**, with longitude and latitude in columns and sites in rows.

1. **Format the metadata table** (see **Figure 2**)

Each dataset has to be accompanied with a table (**DataKey**) that describes **every data entry** with the following information:

- **Matrix**: which matrix does the entry refers to, i.e. any one among comm, traits, envir and coord.
- **Entry**: the spelling of the entry name as it was reported in the four data sheets
- **Variable**: the variable the entry name refers to
- **Unit or factor levels**: the scientific unit, type of variable and/or levels of factor of the variable
- **Description**: brief description of the variable or notes where relevant


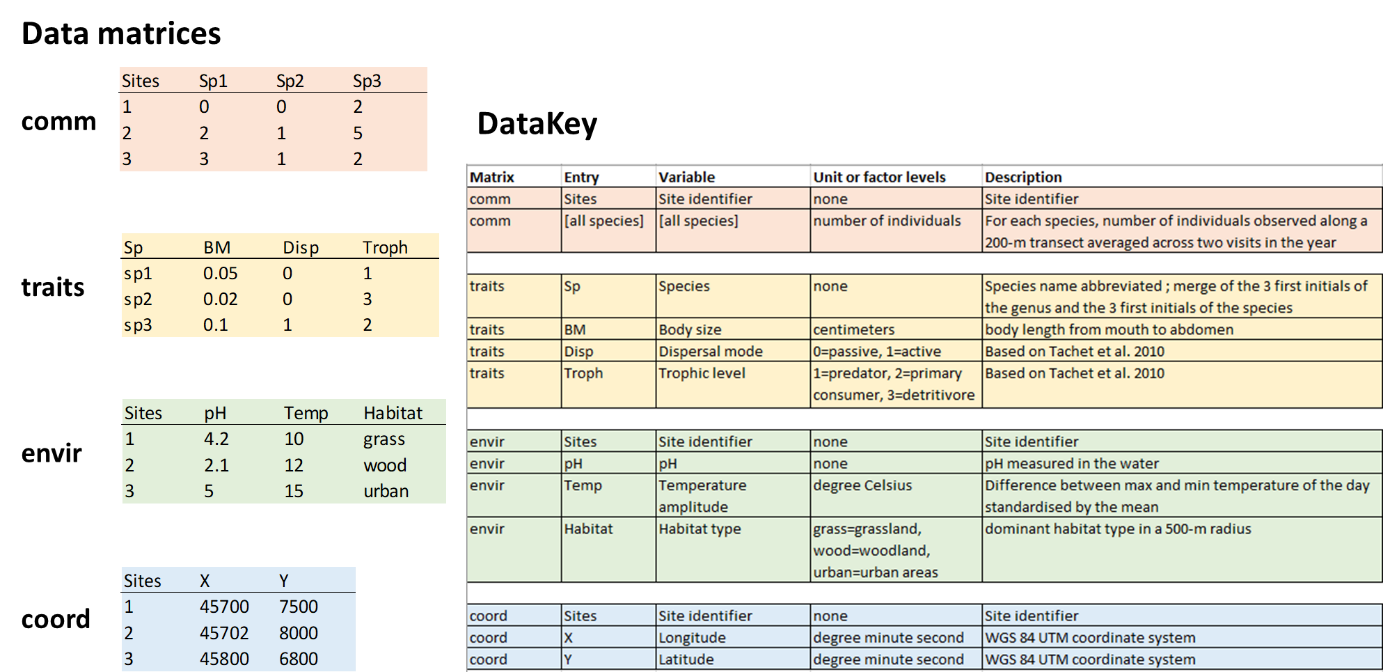


***Figure 2.*** *Example of data matrix structure (Data matrices on the left) and the corresponding metadata information (DataKey on the right)*

1. **Format the species list**

The species list has to report the full name of the taxa that are recorded in the comm data matrix, and any kind of supplementary information that is considered as relevant with respect to the taxonomy of the taxa. Make sure that there is a common column linking comm and splist (see the splist example below; the “TaxCode” column of splist matches with the “Sp” column of comm).

Example:
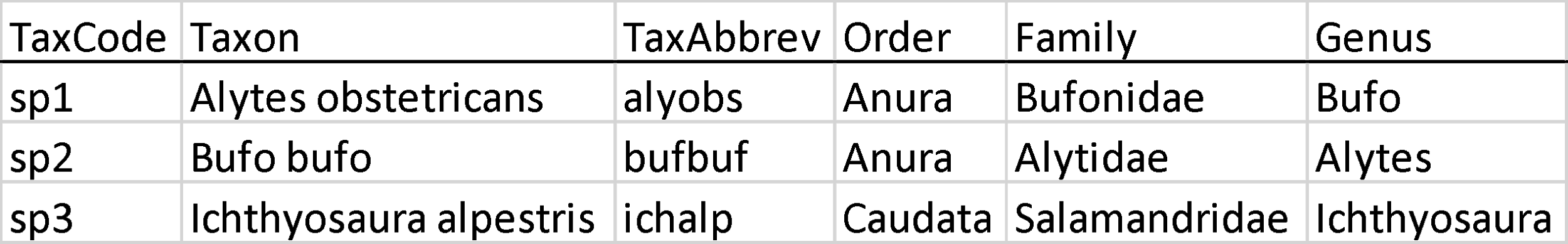


1. **Check the matching across identifiers**

Make sure that:

- site ids match across comm, envir and coord,
- species ids match across comm, traits and splist.

1. **Attach the corresponding publication/report/document**

Make clear what study/publication/document/project the dataset comes from and **how to cite it**.

# Sending the data

Once your dataset has been formatted following the guidelines above, you can send your **file(s) + the pdf of the publication + your affiliation + email contact** via email to the database manager at:

[cestes@idiv.de](mailto:cestes@idiv.de)

# Updating of the database and its citation

If the new dataset fulfils CESTES’ requirements and is provided in the right format with the metadata information, it will be considered for integration to the CESTES database.

The database manager will check and prepare the data for its integration in the database. Once ready, the data will be integrated in the database and uploaded in the iDiv biodiversity data portal^[[1]](#footnote-1)^. This will update the database and generate **a new DOI** for the whole **updated database**, ensuring that the new contributors are acknowledged. Finally, the new contributor will become part of the CESTES consortium via the live project (https://icestes.github.io/).

# Remarks

- If your data:
  - do not have spatial coordinates but have the other three pieces of information,

AND/OR

- - do not have multiple site measurements (i.e. less than 10) but have temporal replicates,

it can be integrated in the non-spatial ancillary section of the CESTES database, ***ceste***.

- If you are willing to share data that include individual-based trait measurements, this will be considered for the extended version of CESTES database that will be the subject of a new promising collaborative project. If interested, please contact the database manager.

For any question, please contact the CESTES database manager at: [cestes@idiv.de](mailto:cestes@idiv.de)

1. https://idata.idiv.de/ [↑](#footnote-ref-1)
